# TGF-β and IL-4 + IL-13 induce neuroplasticity in an *in vitro* model of hPSC-derived sensory neurons

**DOI:** 10.3389/fimmu.2026.1705880

**Published:** 2026-03-03

**Authors:** Carli S. Koster, I. Sophie T. Bos, Chiara Lavitola, Mihaly Balogh, Barbro N. Melgert, Reinoud Gosens

**Affiliations:** 1 Department of Molecular Pharmacology, Groningen Research Institute of Pharmacy, University of Groningen, Groningen, Netherlands; 2 Groningen Research Institute for Asthma and COPD (GRIAC), University Medical Center Groningen (UMCG), University of Groningen, Groningen, Netherlands

**Keywords:** airway inflammation, asthma, cytokine signaling, disease modeling, neuroimmune crosstalk, pluripotent stem cells

## Abstract

Chronic type 2 inflammation is known to drive the neuroplasticity of both afferent and efferent vagal nerves innervating many organs. This results in increased neuronal density and sensitivity, possibly contributing to pathologies such as eczema and asthma. However, the mechanisms driving these neuronal changes, particularly in sensory pathways, remain poorly understood, and appropriate *in vitro* models for their study are lacking. Here, we describe the differentiation of sensory neurons from human pluripotent stem cells. The generation of sensory neurons was validated by verifying the expression of sensory neuron markers, such as β3-tubulin, PGP9.5, TRPV1, Nav1.8, and Piezo1/2, using immunofluorescence, flow cytometry, and RNA sequencing, as well as functional responsiveness to capsaicin using calcium imaging and spontaneous firing using a multi-electrode array. We exposed these hPSC-derived sensory neurons to TGF-β or type 2 cytokines IL-4 and IL-13, both of which play important roles in asthmatic airway remodeling. Both treatments induced neuroplasticity-related changes, such as increased network density and neuronal sensitivity in sensory neurons, albeit more strongly with TGF-β than with IL-4 + IL-13. Our results show robust and reproducible generation of functional hPSC-derived sensory neurons and their usability as a model to investigate the mechanisms underlying neuroplasticity. Furthermore, our findings support a role of TGF-β and type 2 cytokines in the development of neuroplasticity.

## Introduction

The function of multiple internal organs, such as the skin, gut, and lungs, is partially controlled by innervation from the vagal nerve. Chronic type 2 inflammation is increasingly recognized as a driver of peripheral neuroplasticity (1–9). Neuroplasticity is the ability of the nervous system to adapt in response to either intrinsic or external stimuli, resulting in structural, connectivity, and functional changes (10, 11). Evidence from various pathologies, including eczema (3), asthma (4), and allergic rhinitis (12), indicates that persistent inflammation induces the remodeling of both afferent and efferent vagal nerves, resulting in increased neuronal density and sensitivity (3, 4, 6, 10, 12, 13).

Recent studies have identified neuroplasticity as a pathological feature of severe asthma, a disease driven by chronic type 2 inflammation (4, 6). The airways are predominantly innervated by parasympathetic and sensory nerves, with sparse sympathetic innervation. Parasympathetic airway innervation originates from the vagus nerve and is involved in bronchoconstriction, mucus secretion, and coughing (14, 15). Sensory afferent nerves mainly arise from the vagus nerve, with only a small portion arising from the dorsal root ganglia. These dorsal root-derived sensory nerves innervate the large airways, whereas sensory nerves originating from the jugular and nodose vagal ganglia penetrate deeper into the lung tissue (10, 16). The main part of airway sensory neurons originates from the nodose ganglia and is enriched with markers such as VGLUT2, PHOX2B, PIEZO1/2, and several transient receptor potential (TRP) channels involved in autonomic reflex control, mechano- and chemo-sensation (17–19). These nerves have been implicated in chronic type 2 inflammation, through the secretion of sensory neuropeptides, such as substance P, calcitonin gene-related peptide (CGRP) and neurokinin A, that interact with various immune cells (4, 6, 20, 21). Airway sensory afferents constitute both unmyelinated C-fibers and myelinated A-fibers. C-fibers can be subdivided into pulmonary C-fibers, with nerve endings located in the alveoli, and bronchial C-fibers that innervate the airway mucosa (22). C-fibers are characterized by their chemosensitivity to capsaicin, and at high concentrations, small-diameter A-fibers too, through the transient receptor potential cation channel subfamily V member 1 (TRPV1) (22–24). Their stimulation results in rapid shallow breathing, bronchoconstriction, mucus hypersecretion, and coughing, which are all asthmatic symptoms (22, 25, 26).

In general, peripheral sensory neurons are involved in host defense, as the skin, gut, and lung are organs in direct contact with the environment and detect different danger signals, such as heat or cold, cytokines, and mechanical changes (18). In response, they trigger protective effects, such as couching in the lung, itch in the skin, and increased gut motility in the intestines, accompanied by the release of neuropeptides, such as substance P and CGRP, which promote vasodilation and immune cell recruitment (4, 6, 7, 18, 20, 21, 27–31). Modeling neuroplasticity *in vitro* using human disease technologies represents a major unmet need, as animal models lack the translatability to humans. However, human biopsies provide an accurate representation but only provide information on the final stage of neuroplasticity (6). Human pluripotent stem cells (hPSCs) are a novel approach for studying neuroplasticity mechanisms in relation to type 2 inflammation. Previously, our laboratory developed an *in vitro* model to study cholinergic neuroplasticity (9, 13).

In this study, we aimed is to develop an hPSC-derived sensory neuron model and use it to investigate the effects of type 2 inflammation on sensory neuroplasticity. We hypothesized that treatment with transforming growth factor (TGF)-β or type 2 cytokines interleukin (IL)-4 and IL-13 would induce sensory neuroplasticity. Using a previously established protocol for hPSC-derived vagal neural crest spheroids (9), we further differentiated the spheroids into neuronal cultures enriched with sensory neurons. We extensively characterized these sensory neurons using immunofluorescence, gene expression, live-cell Ca^2+^ signaling, and flow cytometry. To model neuroplasticity, we exposed these cells to TGF-β or IL-4 + IL-13 and observed increased network density, increased capsaicin sensitivity, and transcriptional changes mainly related to remodeling and inflammation, compared to untreated sensory neurons. This model provides a foundation for future studies modeling neuroplasticity and neuroimmune interactions in the context of type 2 inflammation, such as that found in asthmatic lungs.

## Methods

### H9WA09 cell culture

H9WA09 cells were obtained from the European Institute for Biology of Ageing (ERIBA, University of Groningen, Netherlands). H9WA09 cells were cultured in mTeSR™ Plus medium (#100-0483, STEMCELL™ Technologies, Canada) in 6-well plates pre-coated with hESC-qualified Matrigel (#354277, Corning, USA). The cells were incubated at 5% CO2 and 37 °C. H9WA09 cells were passaged once the cells became confluent using ReLeSR (#100-0483, STEMCELL™ Technologies, Canada), as described previously (1). The cells were regularly examined for the absence of mycoplasma.

### Generation of sensory neurons from human pluripotent stem cells

**Figure 1:**
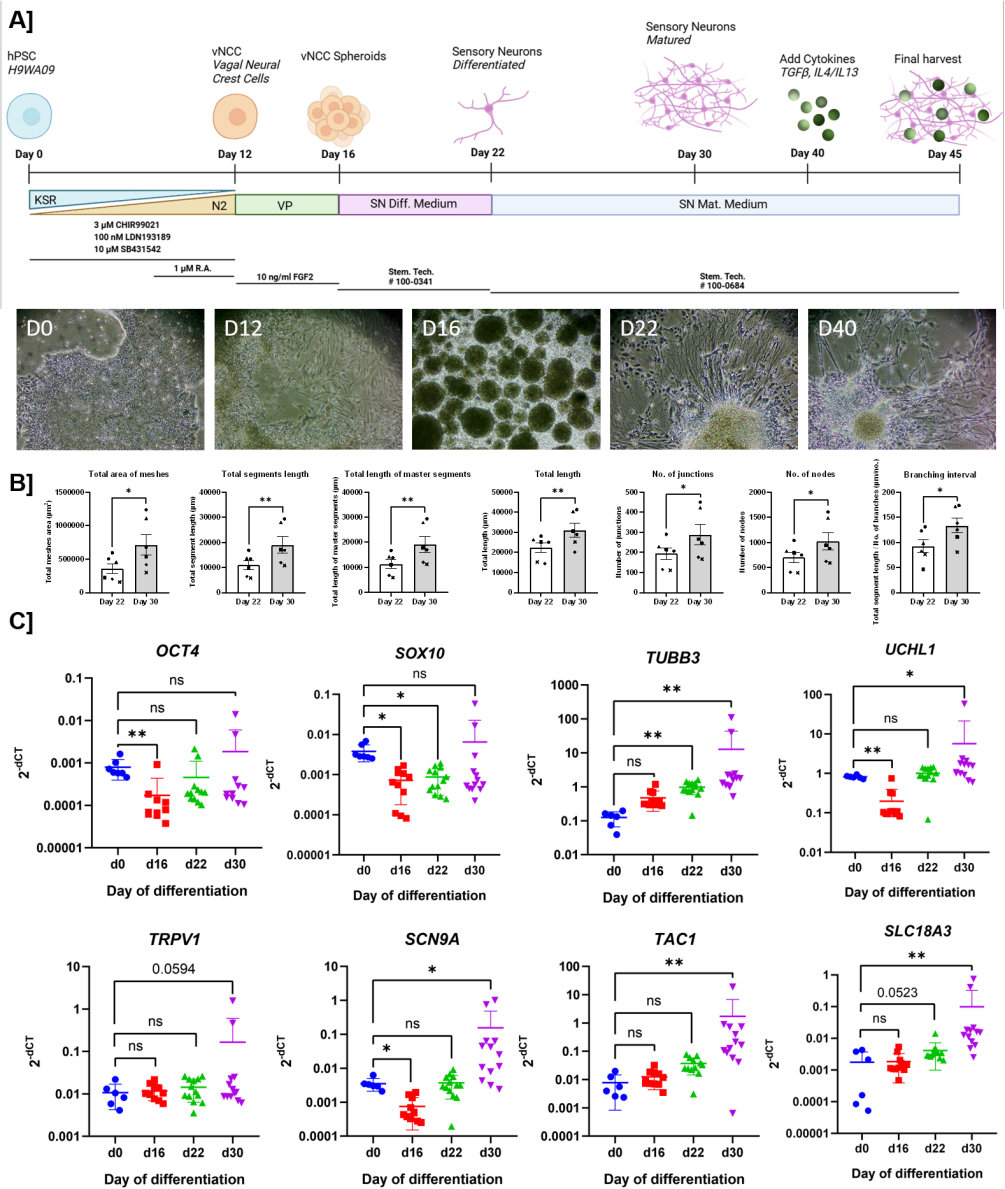
Forty-day differentiation results in the generation of hPSC-derived sensory neurons. **(A)** A schematic representation of the protocol to generate hPSC-derived sensory neurons and 5-day exposure to cytokines TGF-β and IL-4 + IL-13. Created in and adapted from BioRender. Koster, **(C)** (2025) https://BioRender.com/saqr2ek (6). **(B)** Comparing several components of the network density on d22 and d35 shows a clear increase of network density during maturation of the sensory neurons (images acquired using Nikon Ts2 microscope) (N = 6, paired repeated measures one-way ANOVA with Dunnett’s *post-hoc* correction). **(C)** qPCR analysis shows gradual induction of a sensory neuron phenotype, as gene expression levels of several pluripotency, neurons and sensory markers were analyzed on days 0, 16, 22, and 30 (blue, red, green, purple, respectively) to monitor the gradual differentiation (N = 6–13, mixed-effect model with Dunnett’s *post-hoc* correction, performed on log10-transformed data). Levels of significance are indicated as p<0.05 (*), p<0.01 (**), and ns (not significant).

H9WA09 human pluripotent stem cells (hPSCs) were differentiated into vagal neural crest cells (vNCCs), as described previously (9). In short dual SMAD inhibition was induced using SB431542 (10 µM; #72234 STEMCELL™ Technologies, Canada) and LDN193189 (1 µM; #72147, STEMCELL™ Technologies, Canada), and WNT activation was induced using CHIR99021 (3 µM; #72054, STEMCELL™ Technologies, Canada) and retinoic acid (1 µM, #R2625, Sigma-Aldrich, USA). Subsequently, vNCC cells were cultured as floating spheroids for 4 days. Subsequently, vNCC spheroids were plated on Poly-L-ornithine hydrobromide (15 µg/ml; #P3655, Sigma-Aldrich, USA), fibronectin (2 µg/ml; #356008, Corning, USA), and laminin (2 µg/ml; #3400-010-02, Bio-Techne, USA) coated plates and differentiated into sensory neurons using STEMdiff™ Sensory Neuron Differentiation Medium (#100-0341, STEMCELL™ Technologies, Canada) for 6 days. This was followed by 13–15 days of maturation in Sensory Neuron Maturation Medium (#100-0684, STEMCELL™ Technologies, Canada) (**Figure 1A**). The successful differentiation of H9WA09 cells into sensory neurons was validated using immunofluorescence, flow cytometry, PCR, and RNA sequencing to assess the expression of appropriate markers. Live calcium imaging and multi-electrode array (MEA) were used to assess the functionality.

### Treatment of hPSC-derived sensory neurons with cytokines

After maturation of hPSC-derived sensory neurons, the neurons were treated with TGF-β (2 ng/ml; #7754-BH, R&D Systems, USA) or IL-4 (10 ng/ml; #200-04, PeproTech, USA) + IL-13 (3 ng/ml; #200-06, PeproTech, USA), all dissolved in PBS with 0.1% bovine serum albumin (BSA) (#1002695029, Sigma Aldrich, USA) for 5 days. The control samples received the same amount of 0.1% BSA in PBS solution.

### Immunocytochemistry

hPSC-derived sensory neuron samples were fixed in 4% paraformaldehyde (#97H0752, Sigma-Aldrich, USA) for 15 min at room temperature (RT). Subsequently, the cells were permeabilized using 0.3% Triton-X (#101371900, Sigma-Aldrich, USA) for 5 min at RT. A blocking buffer consisting of Cyto-TBS (20 mM Tris base + 154 mM NaCl + 2 mM EGTA + 1 mM MgCl2 in UP, pH = 7.2) + 1% BSA + 2% donkey serum (#017-000-121, Jackson ImmunoResearch, USA) was then applied for 1 h at RT. The cells were then incubated overnight at 4 °C with the primary antibody (**Supplementary Table 1**). The next day, the primary antibody solution was washed away, and the cells were incubated for 1.5 h at RT with the secondary antibody (**Supplementary Table 1**). Finally, the samples were fixed using a mounting medium with DAPI (#ab104139, Abcam, UK), imaged using a Nikon Eclipse Ti2-E/B (Nikon Instruments Inc., USA), and analyzed using ImageJ (National Institutes of Health, USA).

### Network analysis of immunofluorescence stained hPSC-derived sensory neurons

Immunofluorescence images of anti-β3-Tubulin and Donkey-anti-Mouse Alexa Fluor 488 stained hPSC-derived sensory neurons were scanned using an Olympus VS200 Fluorescence Slide Scanner with a PlanXApo ×40/NA = 1.40 oil immersion objective. Images were opened with OlyVIA 3.3 Software, and 10 areas for every batch and condition were selected, and images were cropped for further analysis in ImageJ (National Institutes of Health, USA). The network was analyzed using the Angiogenesis Analyzer plug-in for ImageJ, which is also suitable for neuronal network analysis (32).

### Flow cytometry analysis of hPSC-derived sensory neurons

Flow cytometry analysis was performed on the last day of the experiment. Samples were washed withPBS, twice with 0.5 mM EDTA, and then incubated with EDTA (15 min, 37 °C). The cells were harvested using a serological pipette into 2 mL Eppendorf tubes and centrifuged (290*g*, 1 min, RT). The Fix & PermTM Cell Permeabilization kit (ITK diagnostics, GAS-002) was used according to the manufacturer’s instructions to fix (solution A) and permeabilize (solution B) the cells. Antibody dilutions were prepared in solution B (**Supplementary Table 2**). Staining was performed for 1.5 h per round, either once or for two rounds if a secondary fluorophore-conjugated antibody was required. Final samples were reconstituted in flow cytometry buffer (PBS with 1% BSA, 20mM Glucose (#108342, Merck Millipore, Germany), 2 mM EDTA (#108418, Titriplex^®^ III, SupelCo, SigmaAldrich)) and measured using a NovoCyte Quanteon (Agilent, USA). Data were acquired using NovoExpress Software (Agilent, USA) and analyzed using FlowJo, LLC V10 (Becton Dickinson, BD^®^, USA). First, quality control was performed using FlowAI (33), and the positive reads were exported and compensation was performed prior to the full analysis (**Supplementary Figure 1**).

### Live cell Ca^2+^ imaging using Fluo-4-AM

Live-cell Ca^2+^ imaging was performed to assess neuronal responsiveness. Cells were incubated with Fluo-4-AM (3 µM; #F14201, Invitrogen™, USA) reconstituted in DMSO (#D8418, Sigma), Pluronic^®^ F-127 (0.1%; #P2443, Sigma) in HBSS (#14065-056, Gibco, USA) in the dark for 60 min at RT. Fluo-4-AM was washed off with HBSS before imaging. Data were acquired using a Zeiss LSM 780 NLO (Zeiss, Germany) or Nikon Eclipse Ti2-E/B (Nikon Instruments Inc., USA) by exciting the cells with a 488 nm laser. Images were collected every 500 ms for 3 min. Baseline measurements were performed for 30 s each. After 30 s, the cells were challenged with KCl (100 mM; #104936, SupelCo, SigmaAldrich) or capsaicin (10 nM, 100 nM, 1 μM, and 10 µM; #M2028; Merck, USA). Images were analyzed using ZenBlue (Zeiss LSM 780 NLO) or NIS-Elements software (Nikon Eclipse Ti2-E/B). Neurons were selected for acquisition, and the change in 488 nm light intensity was plotted over time to identify the neuronal response. We corrected for changes in the overall intensity by selecting an area not covered by any cells for background correction. To quantify the neuronal response, the change the change in 488 nm-light intensity from baseline to maximum was measured.

### Multi-electrode array

The spontaneous firing of sensory neurons was measured using A Maestro Pro (Axion Biosystems) MEA system. CytoView MEA 48 plates (Axion Biosystems, M768-tMEA-48W) containing 16 embedded electrodes per well were coated as described in the section “*Generation of sensory neurons from human pluripotent stem cells*.” vNCC spheroids (day 16) were seeded into the center of the MEA well and differentiated on the plate. Repeated recordings of 15 min were made between day 22 and day 50 at 37 °C, 5% CO_2_. Using Axion AxIS Software, spike rates and raw voltages were detected for analysis.

### mRNA isolation of hPSC-derived sensory neurons

After 5 days of exposure to TGF-β or IL-4 + IL-13, both control and cytokine-exposed sensory nerve cultures were harvested using 0.5 mM EDTA, centrifuged (300*g*, 1 min, RT), and the supernatant was aspirated. Cell pellets were stored at −20 °C for future mRNA isolation.

mRNA was isolated using the NucleoSpin RNA XS kit (#740902, Bioké, Leiden, Netherlands) according to the manufacturer’s instructions. RNA was extracted for N = 5 samples for all conditions. RNA concentration was determined using Nanodrop ND-1000. Samples were stored at −80 °C until they were sent for RNA sequencing analysis or RT-qPCR analysis.

### RNA sequencing analysis

Quality control, library preparation, and RNA-sequencing (RNA-seq) analysis were performed by Biomarker Technologies GmbH (BMK, Münster, Germany) using an Illumina NovaSeq 6000 sequencer. The procedure included data quality control, adapter trimming, alignment of short reads, and feature counting. Quality control was performed by BMK, including checking for possible sample and barcode contamination and standard quality metrics using FastQC v0.34 and FastQA. Reads were trimmed for adapter sequences before alignment using Trimmomatic v0.30, and the sample reads were aligned to the Ensembl GRCh38.p14 reference genome. The counts were summarized in count files, that were loaded in RStudio (Rstudio 2023.09.0 + 463; R4.3.1). Subsequently, differential gene expression analysis was performed using DESeq2, prefiltering out any genes with total raw counts across all samples less than 10, and using treatment as the design and batch number as the covariable (~batch + treatment) to allow for paired analysis (34, 35). Gene set enrichment analysis (GSEA) was performed using the fgsea package, applying ranked gene lists based on the DESeq2-results, with gene sets sourced from the MSigDB C5 GO: Biological Processes collection (36).

RNA-Seq files are publicly available at https://www.ncbi.nlm.nih.gov/geo/ using GEO: GSE307973.

### RT-qPCR

For RT-qPCR, cDNA was synthesized using the Reverse Transcription System kit (#A3500, Promega,Netherlands), following the manufacturer’s instructions. qRT-PCR was performed using SYBR Green (#5000840-1250, AMPLIQON, Denmark). A mixture of qPCR forward and reverse primers (1 µM) was used (**Supplementary Table 3**). The program for RT-qPCR reactions started with polymerase activation at 95 °C for 10 min, followed by 45 cycles of PCR cycling: denaturation at 95 °C for 30 s, annealing at 64 °C for 30 s, extension at 72 °C for 30 s, and incubation at 72 °C for 5 min. Subsequently, the melting curves were obtained at 95 °C for 15 s, 55 °C for 15 s, and 95 °C for 15 s. Gene expression analysis was performed using the QuantStudio Real-Time PCR software v1.2 (Thermo Fisher, USA).

### Statistical analysis

For all experiments, GraphPad Prism 8.0 (Dotmatics, USA) was used to compare the effects of treatments using either paired or unpaired (as defined in the figure legend) repeated-measures one-way ANOVA. For RT-qPCR data, groups were compared using log_10_-tranformed data with a mixed-effect model (REML), as batches contained missing data that precluded the use of one-way ANOVA. All statistical analyses were followed by Dunnett’s *post-hoc* test to compare each treatment group with the control group. Prior to the one-way ANOVA and REML analyses, the assumption of normality was assessed using the Shapiro–Wilk test and visual interpretation of QQ-plots. Statistical significance was set at p <0.05.

## Results

### Development and validation of a hPSC-derived sensory neuron protocol

We previously developed a protocol to generate vagal neural crest (vNCC) spheroids from H9WA09 cells through dual SMAD inhibition and early WNT activation for the subsequent development of peripheral cholinergic neurons (9). As airway sensory neurons also originate from vNCC during development (37, 38), we used the initial 16-day protocol to generate vNCC spheroids. These day-16-spheroids were then plated on PLO/FB/LM-coated surfaces, and sensory neuron differentiation medium was used to generate sensory neurons in 6 days, with axonal sprouting and subsequent development of the axons visible within 24 h (**Supplementary Video 1**). On day 22, we switched to sensory neuron maturation medium, and after an additional 2 weeks of culturing, a clear and mature neuronal network was observed (**Figure 1A**). The development of the network was analyzed using brightfield images. This analysis showed significant network development when comparing day 22, the final day of sensory neuron differentiation, to day 30, after two weeks of sensory neuron maturation. There were significantly more segments, master segments, meshes, and branching intervals after maturation on day 30 than on day 22 (**Figure 1B**). An increasing trend was observed in the number of junctions and nodes and the total length of the network, although it did not reach statistical significance.

The sensory neuron phenotype was confirmed by marker expression using PCR and immunofluorescence. PCR analysis also showed gradual formation of the sensory neuron phenotype over the course of differentiation (**Figure 1C**). Gene markers indicative of pluripotency (*SOX10* and *OCT4*) were mainly present on day 0, absent on day 16, and increased again in mature neurons. General neuronal markers (*B3TUB* and *PGP9.5*) gradually increased over time. Sensory-specific markers (*TRPV1, SCN9A, TAC1*) were mainly expressed in the mature network, while having expression levels similar to baseline on day 22. Similarly, an increasing trend in the expression of the cholinergic marker (*SLC18A3*) was also observed over time, indicating that we generated either a mixture of both sensory and cholinergic neurons or that our sensory neurons expressed both sensory and cholinergic markers. This is in line with our flow cytometry data, in which we observed that a substantial proportion (60%) of ChAT^+^ neurons also expressed CGRP (data not shown).

**Figure 2:**
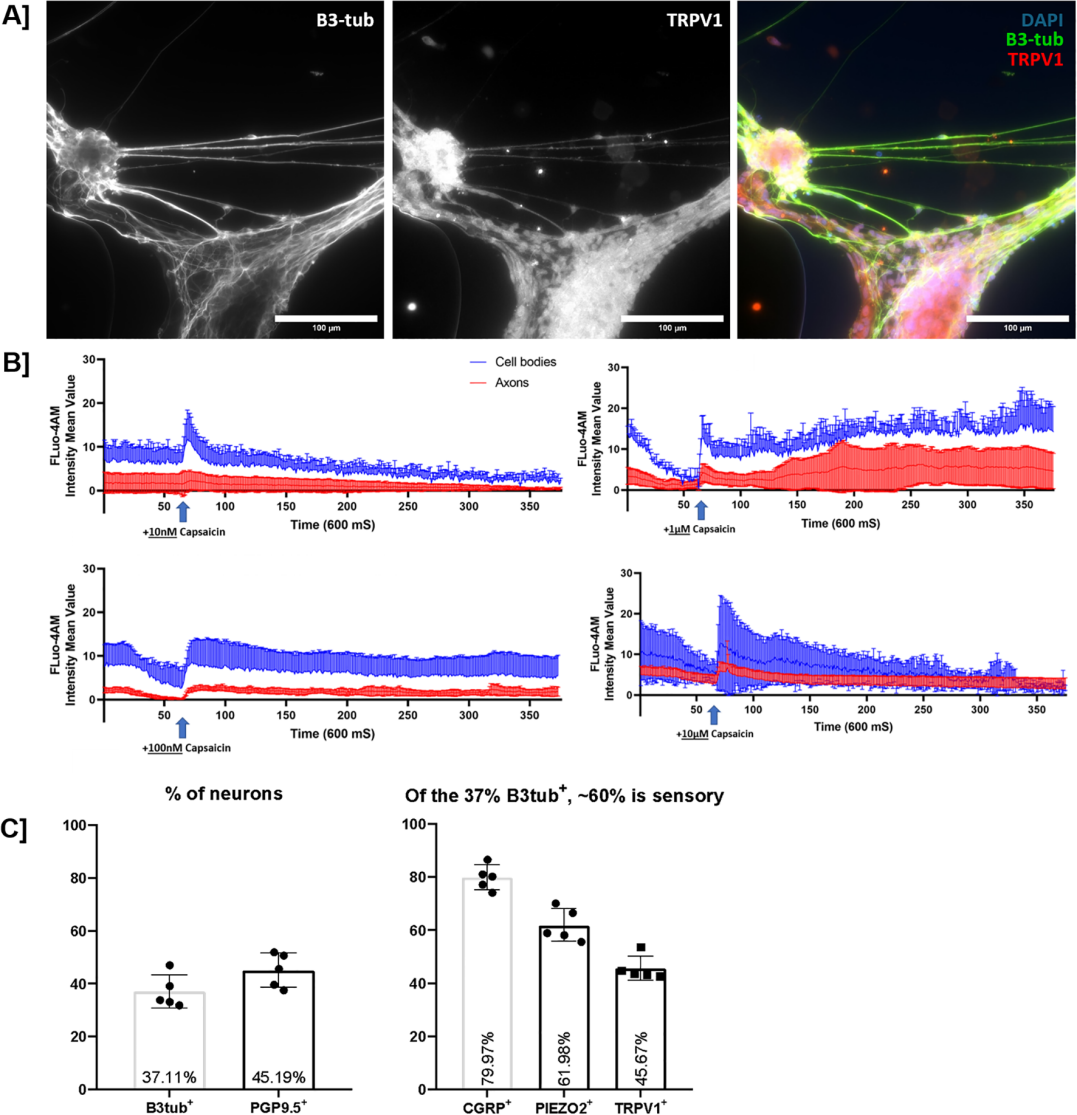
On day 30 mature sensory neurons are generated that express sensory markers and are functional. **(A)** Immunofluorescent staining of hPSC-derived sensory neurons using β3-tubulin confirms the presence of neurons, and TRPV1 confirms the sensory phenotype of the generated neurons (images acquired using the Nikon Eclipse Ti2-E/B with Kinetix camera). **(B)** Capsaicin sensitivity of SN indicates TRPV1 expression and validates the sensory phenotype. Results showing and influx of calcium upon exposure to 10 nM–10 µM of capsaicin. Capsaicin was added after approximately 30 s of imaging, and images were taken every half second for a period of 3 min. AF488 signal was quantified and corrected for background for both 6–10 axons and cell bodies. (Videos acquired using the Zeiss LSM 780 NLO microscope). **(C)** Flow cytometry shows a yield of approximately 40% neurons by β3-tubulin or PGP9.5 expression. Of the 37.11% β3-tubulin^+^ cells, 80% expressed CGRP, 62% PIEZO2, and 46% TRPV1.

Neuronal network formation after 40-days was confirmed using β-3-tubulin staining to show the presence of neurons and TRPV1 staining to confirm the presence of a sensory phenotype in the network (**Figure 2A**). Next, we investigated the functionality of sensory neurons using fluo4-AM calcium imaging by exposing the neurons to capsaicin (TRPV1 agonist) (23, 24). We assessed their sensitivity to a sensory neuron-specific stimulus and found that sensory neurons reacted in a concentration-independent manner (**Figure 2B**). Responses were measured in both the axons and cell bodies, although they were stronger in the cell bodies. Functional TRPV1 activity and the responsiveness of sensory neurons indicate nociceptor-like properties. Additionally, MEA data showed spontaneous firing of the neurons starting around day 35 and continuing until day 50, indicating this as the ideal time frame for further experiments (**Supplementary Figure 2**). Flow cytometry was used to confirm marker expression and determine the yield of differentiation. The overall neuronal yield of ~41% was determined using β-3-tubulin (37%) and PGP9.5 (45%) staining. Of the β3-tubulin^+^ cells, the majority expressed sensory markers: CGRP^+^ (80%), PIEZO2^+^ (62%), and TRPV1^+^ (46%) (**Figure 2C**).

**Figure 3:**
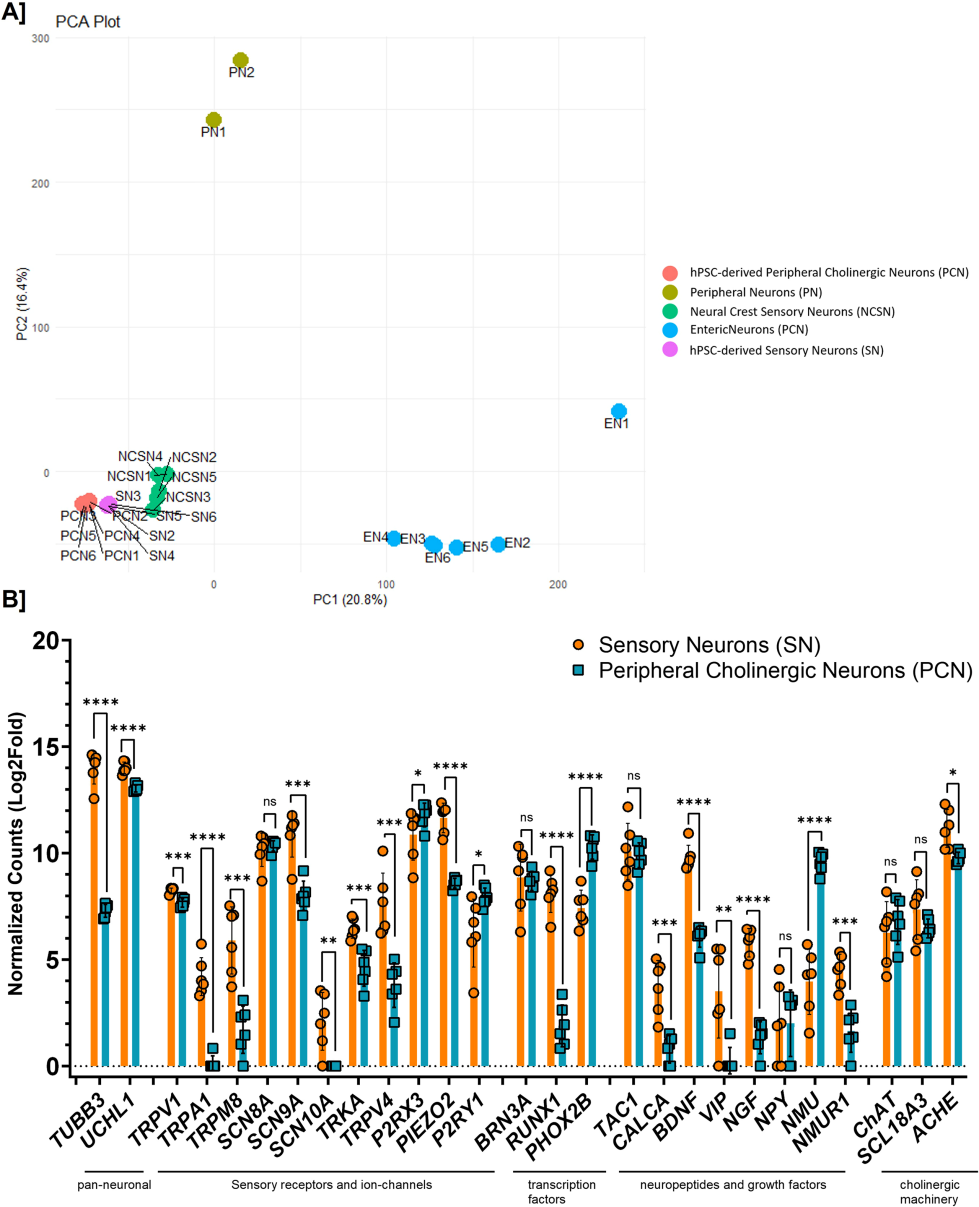
hPSC-derived sensory neurons show a distinct but reproducible genotype. **(A)** Principal component analysis of DESeq2 data using several publicly available neuronal datasets, and two hPSC-derived neuronal subtypes from our laboratory, separated on PC1 vs. PC2. Reveals genotypical similarities of our hPSC-derived sensory neurons to neural crest sensory neurons. **(B)** Gene expression analysis comparing our sensory-enriched airway neurons (SN) and peripheral cholinergic neurons (PCN) show an increased presence of a sensory phenotype. Levels of significance are indicated as p<0.05 (*), p<0.01 (**), p<0.001 (***), p<0.0001 (****), and ns (not significant).

To further investigate the sensory phenotype, we performed RNA-seq analysis and principal component (PA) analysis, comparing our sensory neurons to other neuronal datasets (**Figure 3A**). Our sensory neuron dataset most resembled neural crest sensory neurons (39), confirming the enrichment of the sensory phenotype. The similarity to peripheral cholinergic neurons can be attributed to the overlapping origin of vagal neural crest progenitors (9). The hPSC-derived peripheral neuron (40) and enteric neuron (41) datasets were further removed from the PC analysis, indicating less transcriptomic resemblance, strengthening our hypothesis that we have enriched for sensory neurons. Additional comparisons of our sensory neurons with parental H9WA09 cells (42), cholinergic neurons, and murine dorsal root and nodose ganglia datasets (was obtained from the Gene Expression Omnibus: GSE190499) suggested that our neurons expressed a pulmonary sensory neuron phenotype based on the expression of *SLC17A6, TRPV1, TRPA1, PIEZO1/2, TAC1, CALB1, NPY1R, IL6*, and *KCNG1* (**Supplementary Figure 3**) (17–19).

We then compared our sensory neurons with a previously generated peripheral cholinergic neuron dataset (**Figure 3B**). The expression of both pan-neuronal markers β-3-tubulin (*TUBB3*) and PGP9.5 (*UCHL1*) was higher in sensory neurons, suggesting either a higher overall yield or that neurons were more enriched for *TUBB3* and *UCHL1*. We also investigated the gene expression of several sensory neuron markers and ion channels, including *TRPV1*, *TRPV4*, TRP cation channel subfamily A member 1 (*TRPA1*), *TRPM8*, sodium voltage-gated channel alpha subunit 8 (*SCN8A*), *SCN9A*, *SCN10A*, neurotrophic receptor tyrosine kinase 1 (*TRKA*), purinergic receptor P2X3 (*P2RX3*), purinergic receptor P2Y1 (*P2RY1*), and piezo type mechanosensitive ion channel component 2 (*PIEZO2*). The expression of most of these genes was higher in sensory neurons than in peripheral cholinergic neurons, except for *SCN8A*, *P2RX3*, and *P2RY3*. The transcription factor BRN3A (*POU4F1*, POU domain, class 4, transcription factor 1) was not differentially expressed between the two neuron types, but RUNX family transcription factor 1 (*RUNX1*) and transcription factor paired-like homeobox 2b (*PHOX2B*) expression was significantly higher in sensory neurons. For neuropeptides and growth factors, expression was significantly higher in sensory neurons, except for tachykinin precursor 1 and neuropeptide Y, for which no significant changes were observed, and neuromedin U (*NMU*), for which expression was significantly higher in peripheral cholinergic neurons. The expression of cholinergic markers, choline O-acetyltransferase (*CHAT*) and vesicular acetylcholine transporter (VAChT, *SLC18A3*), was similar in both neuron types, whereas acetylcholinesterase (*ACHE*) was higher in sensory neurons. Overall, the enriched expression of sensory markers and ion channels highlights the sensory identity of our model, providing a suitable foundation for investigating sensory neuroplasticity.

### Effects of TGF-β and IL-4 + IL-13

After characterization of our sensory neuron cultures, we exposed neurons to TGF-β (2 ng/ml) or a combination of IL-4 (10 ng/ml) and IL-13 (3 ng/ml) (25, 43, 44). TGF-β can play both pro- and anti-inflammatory roles, with important implications for type 2 inflammation in asthma, where it is strongly associated with airway remodeling (45–47). IL-4 and IL-13 are central cytokines in type 2 inflammation, and both bind to the IL-4Rα receptor (48). To assess the neuroplasticity of sensory neurons in response to these treatments, we studied RNA-seq transcriptional changes, in addition to analyzing network density and sensitivity of the neurons using β-3-tubulin^+^ network analysis and Ca^2+^-sensitivity measurements. RNA-seq analysis of the log2-normalized counts of the TGF-β, IL-4, and IL-3 receptors showed expression of all receptors, with no changes upon treatment, except for a slight increase in IL13RA2 expression after IL-4 + IL-13 treatment (**Supplementary Figure 4**).

**Figure 4:**
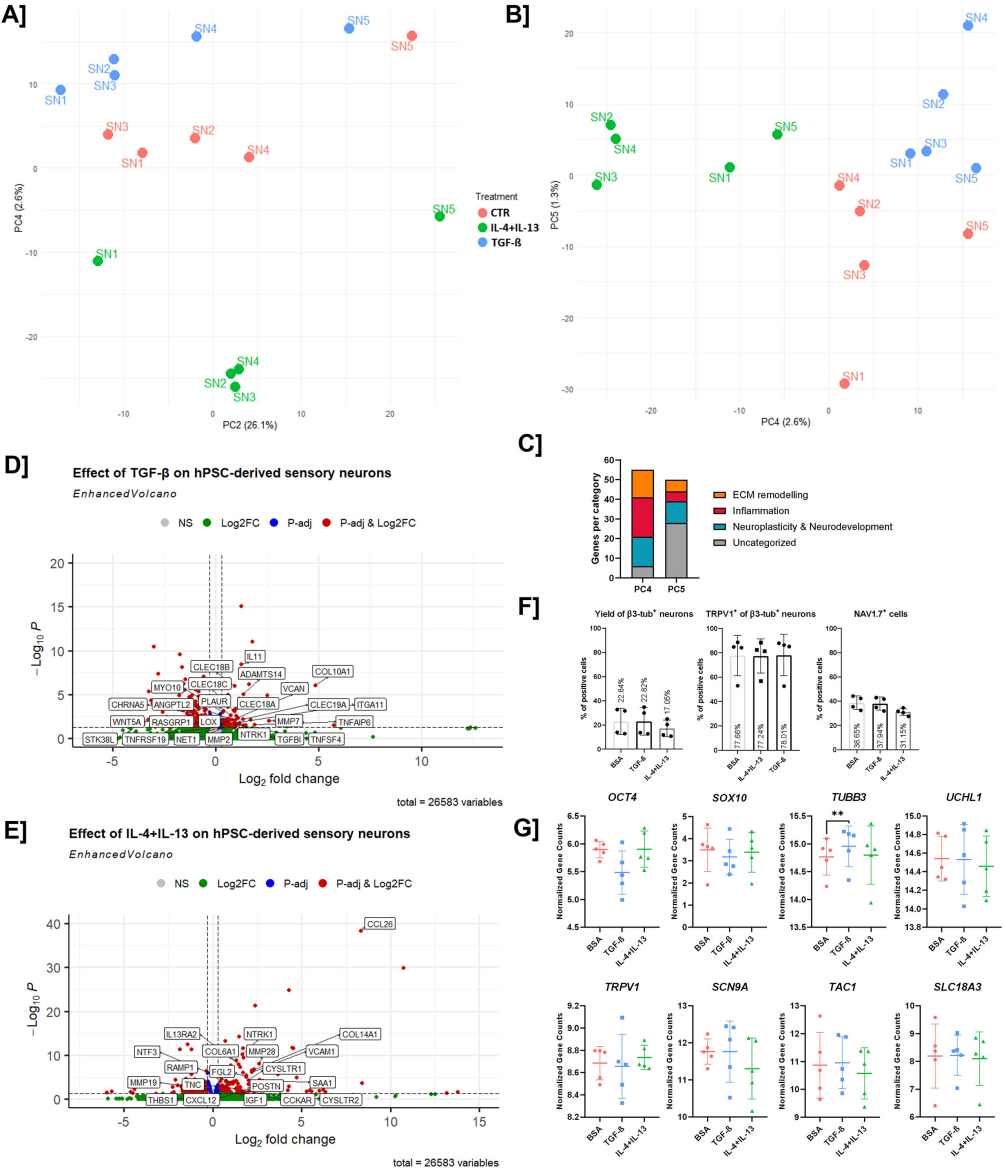
Five-day exposure to TGF-β or IL-4+IL-13 results in specific genotype shifts but does not induce differentiational changes. **(A)** Principal component (PC) analysis plot showing the dispersion of the control (CTR) hPSC-derived sensory neurons, compared to IL-4 + IL-13 and TGF-β treated cells. Separated in PC2 vs PC4 **(A)**, or PC4 vs PC5 **(B)**. **(C)** Categorization of the genes determining PC4 and PC5 separation, indicating changes in the areas of ECM remodeling, inflammation, and neuroplasticity. **(D)** Volcano plot showing differentially expressed genes (DEGs) in TGF-β-treated sensory neurons vs CTR **(D)** or IL-4 + IL-13-treated sensory neurons vs CTR **(E)**, highlighting key upregulated and downregulated genes. Genes were identified using DESeq2 with an adjusted p-value <0.05 and |log_2_FC| >1 as significance thresholds. **(F)** Flow cytometry analysis showed no significant changes in yield of sensory neurons after treatment with cytokines (N = 5, one-way ANOVA with Dunnett’s *post-hoc* correction). **(G)** qPCR analysis shows no changes in several pluripotency, neurons, and sensory markers, as was observed over-time during the differentiation from days 0 to 30 (N = 5, one-way ANOVA with Dunnett’s *post-hoc* correction). Levels of significance are indicated as p<0.01 (**).

PC analysis of transcriptomic data revealed separation of TGF-β- and IL-4 + IL-13-treated sensory neurons compared to control in PC2 vs PC4 (**Figure 4A**), as well as in PC4 vs PC5 (**Figure 4B**). The separation observed in PC1, PC2, and PC3 was caused by the batch effect ofdifferentiation instead of the treatment, indicating a small transcriptional impact of both treatments compared to the variation between batches of differentiation. Subsequently, the top 50 genes driving the separation of PC4 and PC5 were extracted (**Supplementary Table 4**). Several genes involved in the separation of PC4 and PC5 are linked to changes in extracellular matrix (ECM) remodeling (*COL14A1, COL6A5, COL6A6, COL10A1, POSTN, VCAM1, MMP28, ITGA11, ASPN, ITIH2, ENPP1*), inflammation (*ALOX15, IL6, IL13RA1, CCL26, TNFSF10, CXCL6, TNFAIP6, SAA1, ITIH2*), and neuroplasticity and neuronal development (*CD44, HMGA2, KCNJ2, GNG4, GPR50, CARTPT, DBH, GNRH1, MOSMO*) (**Figure 4C**). Interestingly, PC analysis indicated that treatment with TGF-β or IL-4 + IL-13 induced distinct transcriptional changes, as both treatments were separated from both the control condition and each other.

A more in-depth investigation of the impact of both treatments on differentially expressed genes (DEGs) confirmed that treatment with TGF-β (**Figure 4D**) or IL-4 + IL-13 (**Figure 4E**) induced different expression patterns. Sensory neurons treated with TGF-β upregulated genes in pathways similar to those found for PC4 and PC5 compared to untreated sensory neurons, such as neuroplasticity and neurodevelopment (*WNT5A, NET1, MYO10, STK38L, VCAN, LOX, PLAUR, NTRK1, RASGRP1, CHRNA5*), ECM remodeling (*COL10A1, ITGA11, TGFBI, VCAN, LOX, PLAUR, MMP2, MMP7, ADAMTS26, ADAMTS14*), and inflammation (*TNFAIP6, IL11, TNFSF4, TNFRSF19, CLEC19A, CLEC18A/B/C, ANGPTL2*). After treatment with IL-4 + IL-13, differentially expressed genes were identified compared to untreated sensory neurons. These differentially expressed genes differed when compared to TGF-β treatment, though they did fall in similar categories, namely neuroplasticity and neurodevelopment (*NTRK1, NTF3, CXCL12, CCKAR, RAMP1, LDDB, IGF1*), ECM remodeling (*COL14A1, COL6A1, POSTN, THBS1, TNC, MMP28, MMP19*), and inflammation (*CCL26, IL13RA2, VCAM1, CYSLTR1, CYSLTR2, SAA1, FGL2*).

**Figure 5:**
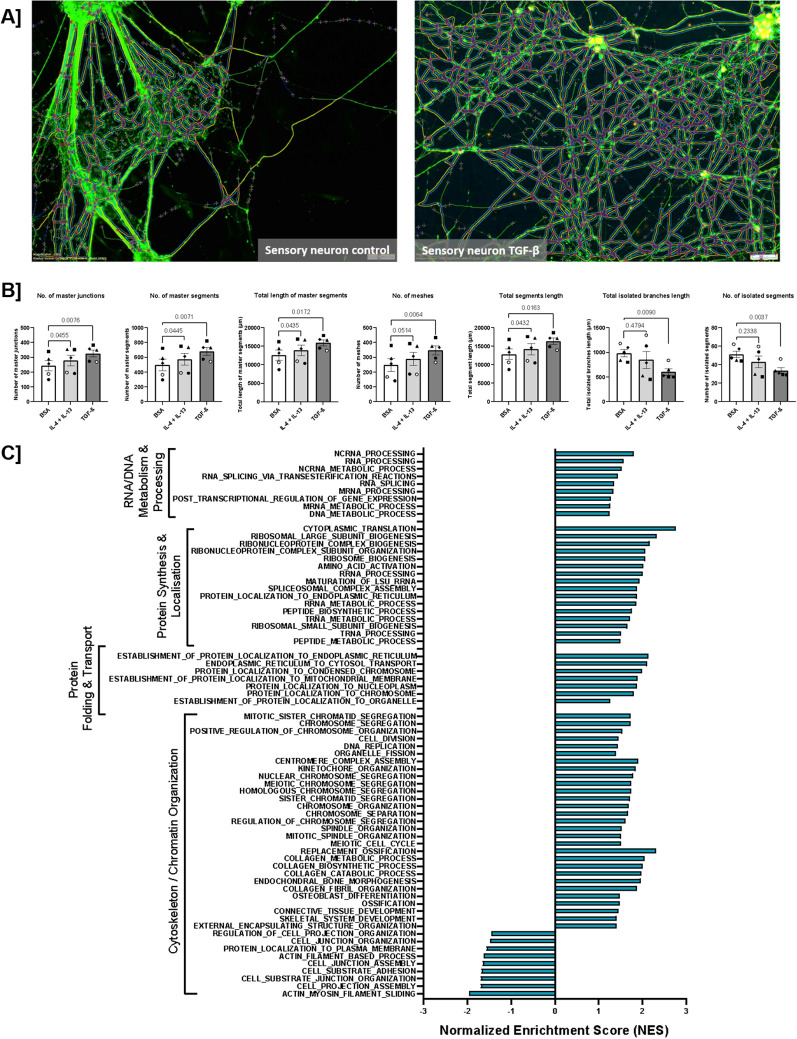
TGF-β and IL-4 + IL-13 induce neuroplasticity-like changes in the formed sensory neuron network. **(A)** Sensory neuron networks, both control and exposed to TGF-β or IL-4 + IL-13, were stained with β3-tubulin. (Images acquired using the Nikon Eclipse Ti2-E/B with Kinetix camera). **(B)** Network analysis was performed using the ‘Angiogenesis Analyzer for ImageJ.’ Exposure to TGF-β more so than IL-4 + IL-13, induced an increase in the overall network density (N = 5, paired repeated measures one-way ANOVA with Dunnett’s *post-hoc* correction). **(C)** GSEA analysis of TGF-β exposure on network density-related biological processes.

**Figure 6:**
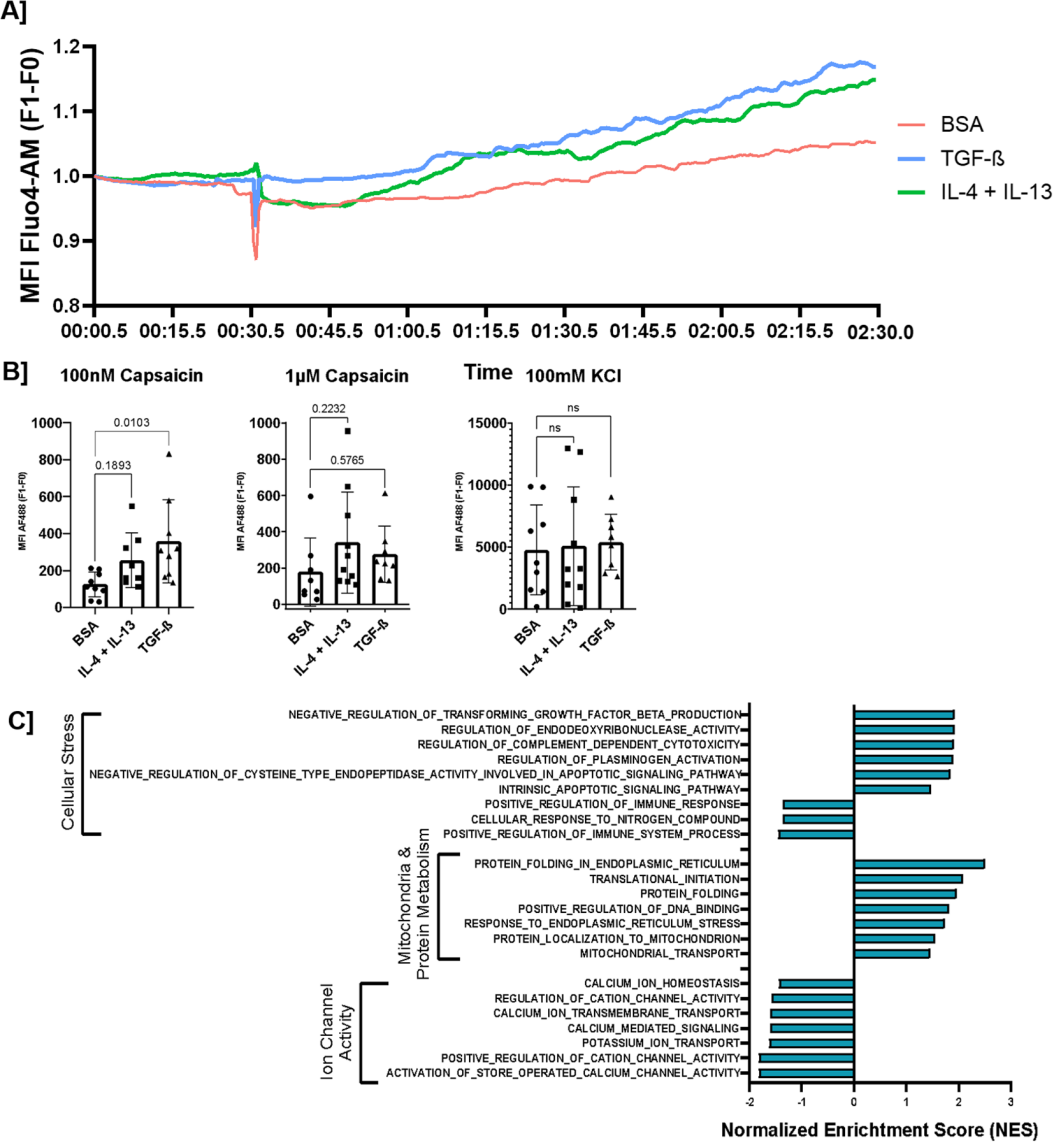
Exposure to TGF-β and IL-4 + IL-13 increased sensitivity to 100 nM capsaicin for hPSC-derived sensory neurons. **(A)** An example of the Fluo4 measurement for CTR and TGF-β-, or IL-4 + IL-13-treated sensory neurons over time, with exposure to capsaicin at 30 s. Measurements are controlled for the first time point (F1/F0). (Images acquired using the Nikon Eclipse Ti2-E/B with Kinetix camera.) **(B)** hPSC-derived sensory neurons were exposed to capsaicin, an agonist of the TRPV1 receptor, or kaliumchloride (KCl). The reaction to 100 nM and 1 µM capsaicin was quantified and compared between hPSC-sensory neurons exposed to IL-4 + IL-13, TGFβ, or control. (N = 5, paired repeated measures one-way ANOVA with Dunnett’s *post-hoc* correction). **(C)** GSEA analysis of TGF-β exposure on network sensitivity-related biological processes.

Subsequently, we performed gene set enrichment analysis (GSEA) for both treatments. In this analysis, the distinction between the biological pathways induced by both treatments became most apparent. After TGF-β treatment, biological processes linked to increased network formation were identified (**Figure 5C**). Pathways related to increased RNA/DNA metabolism and processing, as well as increased protein synthesis, folding, and transport, were also detected. More specifically, many biological processes linked to the development of the cytoskeleton and microtubules were observed, which are essential for the development of axons in a neuronal network. Moreover, after TGF-β treatment, several changes were evident in biological processes that could be linked to increased Ca^2+^ sensitivity of the sensory neurons (**Figure 6C**). We identified an increase in multiple stress-related processes and mitochondrial activity. For ion channels, we found both a decrease in calcium channel activity, indicating reduced sensitivity, and a decrease in the activity of potassium channels, indicating impaired hyperpolarization, which could result in increased sensitivity (49). Additional GSVA indicated increased downstream signaling through SMAD2/3 and no significant changes in SMAD-independent signaling after TGF-β treatment (**Supplementary Figure 5**). After IL-4 + IL-13 treatment, we observed fewer changes that could be linked to neuroplasticity. Related to network density, we found increased mitochondrial and protein metabolism, but mainly a downregulation of biological processes linked to axon development (**Supplementary Figure 6**). In the area of neuronal sensitivity, both mitochondrial activity and stress response were increased, similar to what was observed for TGF-β treatment (**Supplementary Figure 7A**). In addition, several pathways linked to neuroimmune interactions were upregulated. However, synaptic development and activity (**Supplementary Figure 7A**), as well as most ion channels, were found to be downregulated (**Supplementary Figure 7B**).

Based on these transcriptomic data, showing differential expression in areas of extracellular matrix and neuroplasticity, and the GSEA, showing upregulation of biological processes that can be linked to increased network density, we investigated changes in the neuronal network after exposing mature sensory neurons to TGF-β or IL-4 + IL-13 for 5-days. We found significant changes in the network for both treatments with TGF-β and IL-4 + IL-13, with the effect of TGF-β being the most pronounced. Our data show an increase in the number of junctions, segments, and meshes, as well as an increase in the overall sum of the length of the network (**Figures 5A, B**), with the effects of TGF-β treatment being consistently larger than those of IL-4 + IL-13. Accordingly, the length of the isolated branches and the number of isolated segments were lower, indicating a more expanded and developed network than the untreated sensory nerve network. To further investigate these findings, we compared the expression of β3-tubulin and the sensory markers TRPV1 and NAV1.7 using flow cytometry (**Figure 4F**). Interestingly, these data indicated no changes in the yield of either overall neurons or sensory neurons specifically after TGF-β or IL-4 + IL-13 treatment. Similarly, PCR analysis of several genes linked to sensory neuron differentiation showed no significant changes, except for *TUBB3*, which was higher following TGF-β treatment than in the control (**Figure 4G**).

Subsequently, we investigated the changes in the firing sensitivity of sensory nerves. We used both 100 nM and 1 µM capsaicin to specifically measure the firing sensitivity of TRPV1^+^ sensory neurons. Our data show that both IL-4 + IL-13 and TGF-β treatments increased the firing sensitivity of the sensory neuron population (**Figures 6A, B**). For 100 nM capsaicin, we found a significantly higher firing sensitivity in response to TGF-β treatment and a trend of higher firing sensitivity after IL-4 + IL-13 treatment. For 1 µM capsaicin, there was a similar trend, but this was not statistically significant for either TGF-β or IL-4 + IL-13 treatment. The response to potassium chloride, a general depolarizer of neurons and a non-specific stimulus for sensory or cholinergic populations (50), did not induce statistically significant changes in responsiveness after cytokine exposure.

## Discussion

In this study, we established a differentiation protocol for peripheral sensory neurons. Additionally, we aimed to test whether the growth factor TGF-β and/or type 2 cytokines IL-4 and IL-13 induce sensory neuroplasticity. To do so, we treated hPSC-derived sensory neurons with TGF-β or IL-4 + IL-13 for 5 days. In general, TGF-β induced stronger effects than IL-4 + IL-13; it induced transcriptional changes in microtubule formation, mitochondrial activity, and neuronal hyperpolarization, further confirmed by an increased β-3-tubulin^+^ network and enhanced capsaicin-specific sensitivity. IL-4 + IL-13 treatment had less effect at the transcriptional level, even though a denser network and a trend toward higher capsaicin sensitivity were observed.

The sensory neuron differentiation protocol was based on a previously established method for developing vNCCs precursors (9). As the vagus nerve is the primary source of airway innervation, providing both afferent sensory and predominantly cholinergic efferent nerves, the developmental origin of the model provides a relevant framework for modeling airway innervation (51). Transcriptomic profiling revealed that our sensory neurons expressed a marker set associated with pulmonary sensory innervation, including *SLC17A6, TRPV1, TRPA1, PIEZO1/2, TAC1, CALB1, NPY1R, IL6*, and *KCNG1*, and minimal expression of *GLP1R and GABRA1* (17–19). In comparison to murine dorsal root and nodose ganglia nociceptors, H9WA09 cells, and hPSC-derived cholinergic neurons, our sensory neurons showed the strongest expression of this marker set. Although *GLP1R* and *GABRA1* were also strongly expressed, their presence in murine airway neurons suggests that they are not definitive discriminators. Kornfield et al. (52) reported that airway-innervating sensory neurons involved in cough express PIEZO2, TRPV1, TAC1, Nav1.7, and Nav1.8, suggesting that Nav1.7 and Nav1.8 are expressed in addition to the pulmonary-specific marker set (52), even though pulmonary sensory neurons are more tailored towards mechano- and chemosensation rather than pain detection. These markers, together with sensory-specific neuropeptides CGRP, substance P and vasoactive intestinal peptide, involved in neuroimmune signaling, were transcriptionally evident in our neurons, supporting a sensory phenotype (20, 29, 51). Moreover, these sensory markers were more abundant in our neurons than in peripheral cholinergic neurons, confirming sensory enrichment using this protocol. Nevertheless, this molecular profile is not exclusive to pulmonary-innervating sensory neurons and overlaps with that of other peripheral nociceptive and vagal neuronal subtypes. Therefore, the ability to generate pulmonary-specific sensory neurons is limited. Consistent with this, while immunofluorescence and flow cytometry showed a sensory phenotype (TRPV1^+^/Nav1.8^+^), approximately 60% of neurons also expressed cholinergic markers (ChAT^+^/VAChT^+^) (9). Such overlap of markers is in line with the accumulating evidence that cholinergic marker expression is not restricted to efferent neurons but is also present in subsets of peripheral sensory neurons across species (53–56). Overall, the generated mixed phenotype did not indicate deviating differentiation outcomes but reflected the heterogeneity representative of the vagal neuronal diversity of the lung. Although we cannot claim that our model specifically generates only pulmonary-innervating sensory neurons, it does provide a human sensory-enriched vagal neuronal model suitable for studying cytokine-induced neuroplasticity. Therefore, the observed structural, functional, and transcriptional changes should be interpreted as mechanisms broadly applicable to peripheral sensory neurons in inflammatory environments, instead of being limited to airway-innervating sensory neurons.

Various protocols for successfully differentiating hPSCs into sensory neurons have been published, but they often focus on pain perception research (57–60). These previous studies reported yields varying between 60% and 90% of either pan-neuronal markers without specific determination of a sensory marker yield or quantifying the yield using only one sensory marker. STEMCELL™ Technologies reported a yield of approximately 90% neurons and approximately 25% of cells positive for BRN3A using a similar protocol (61). We determined the yield using flow cytometry and confirmed approximately 45% neuronal cells, of which 60%–80% expressed various sensory markers (TRPV1, NAV1.8, and PIEZO2). The expression of these markers was confirmed using immunofluorescence and PCR. To our knowledge, this is the first hPSC-derived sensory neuron model used to study airway neuroplasticity in type 2 inflammation. Our flow cytometry data of multiple independently generated batches of sensory neurons showed consistent expression of pan-neuronal markers β-3-tubulin and PGP9.5 and sensory markers CGRP, NAV1.7, and TRPV1, indicating that our protocol generates a reproducible neuronal population. PC analysis further confirmed the transcriptomic similarity of our sensory neurons to hPSC-derived neural crest sensory neurons (39). The similarity to hPSC-derived peripheral cholinergic neurons likely reflects their common vagal neural crest origin (9). Many previously established protocols have reported limited excitability or physiologically relevant firing (57). Using a Fluo4-AM based Ca^2+^ sensitivity assay, we confirmed the functional activity of our sensory neurons in response to the general stimulus KCl and the sensory-specific stimulus capsaicin (23, 24). We showed responses across a capsaicin concentration range (10 nM–10 µM), albeit not dose-dependent, suggesting that even lower concentrations could suffice, especially since non-specific activation has been reported at ≥10 µM (62). In addition to Fluo4-AM-based Ca^2+^-based sensitivity assays, functionality can also be assessed using MEA, which provides complementary electrophysiological data. Odawara et al. (63) reported capsaicin sensitivity in their induced pluripotent stem cell-derived sensory neurons using MEA, in line with the spontaneous firing detected in our neurons between days 30 and 57 using MEA. Another study showed responsiveness to 100 nM capsaicin in stem cell-derived sensory neurons, similar to dorsal root ganglia-isolated neurons responding to 10 nM–1 µM capsaicin (64), further validating the concentration range and physiological relevance of our model. Altogether, these findings highlight the functional maturity and physiological relevance of our sensory neuron model, which exhibits functional TRPV1 expression and capsaicin-specific responses.

Next, we aimed to test whether TGF-β and/or IL-4 + IL-13 induce neuroplasticity using our hPSC-derived sensory neuron model. Neuroplasticity is a pathological feature of several pathologies including eczema, Crohn’s disease and asthma, observed in both human and allergen-sensitized mice (4–6, 9, 13). In asthma, TGF-β plays an important role as a key driver of chronic airway remodeling, including airway basement membrane thickening, subepithelial fibrosis, airway smooth muscle hypertrophy and hyperplasia, and epithelial remodeling, resulting in airway wall thickening (45–47, 65). IL-4 and IL-13, signature type 2 cytokines, drive immune cell recruitment and activation, such as eosinophil recruitment, IgE class switching, and mucus secretion across a range of type 2 inflammatory diseases (46, 48). As both bind to the IL-4Rα/IL-13Rα1 heterodimer receptor, they are commonly studied together (66). RNA-seq analysis indicated the expression of all TGF-β, IL-4, and IL-13 receptors, indicating that the sensory neuron model possesses the ability to respond to the treatments and study cytokine-induced neuroplasticity. Transcriptome analysis and GSEA results of TGF-β-treated sensory neurons revealed upregulation of pathways linked to microtubule formation, protein metabolism, and ECM remodeling, all of which are required for neurite outgrowth, development, and maintenance of a denser neuronal network (67), as confirmed by our β-3-tubulin^+^ network analysis. These transcriptional findings are consistent with the activation of canonical TGF-β signaling via SMAD2/3, which is known to regulate extracellular matrix production, cytoskeleton organization, and neuroplasticity (68–71). Together, these processes support the structural remodeling required for neurite extension. Although SMAD-independent pathways, such as MAPK, PI3K-AKT, and RhoA-ROCK signaling, have been previously linked to cytoskeletal remodeling, protein metabolism, and neuronal plasticity (72–74), they were not significantly upregulated. They may still contribute by supporting increased energy and biosynthesis demands to sustain neuronal remodeling. Together, these findings indicate that TGF-β-induced sensory neuronal plasticity in this model is likely mediated mainly by SMAD-dependent downstream signaling, and a less clear but not absent effect of SMAD-independent downstream signaling is observed.

In contrast, IL-4 + IL-13 treatment did not induce strong transcriptional activation of pathways associated with a denser neuronal network. Consistently, the β-3-tubulin^+^ network was less dense following IL-4 + IL-13 treatment. Additionally, flow cytometry indicated no significant change in the overall neuron yield after either treatment, suggesting that the effects stem from enhanced network complexity and longer neurites rather than increased differentiation. This was supported by the absence of upregulation of neuronal differentiation pathways. Overall, these findings indicate that TGF-β and IL-4 + IL-13 promote neuroplasticity by increasing network density and neurite extension (4, 13).

Besides increased neuron density and neurite length, increased neuron sensitivity is also a feature of neuroplasticity. The transcriptome and GSEA data for both treatments showed upregulation of genes involved in mitochondrial activity and stress responses, which affect Ca^2+^ signaling and sensitivity (75, 76). Furthermore, both calcium and potassium channel-related pathways were downregulated. We confirmed the relevance of these pathways using Ca^2+^-based sensitivity assays, which showed increased sensitivity to 100 nM capsaicin. The absence of increase at 1 µM capsaicin may result from TRPV1 receptor saturation at higher concentrations (62). Additionally, the absence of increased sensitivity to KCl suggests that the neuroplasticity effect is sensory neuron-specific, instead of changing overall neuronal sensitivity (23, 24). These findings suggest a stronger effect of the downregulation of potassium than that of calcium channels. Potassium channels normally contribute to membrane hyperpolarization and limit excitability, resulting in impaired repolarization, which can lower the threshold for activation (77). This enhances neuronal responsiveness despite decreased calcium channel expression. Overall, the stronger effects after TGF-β treatment compared to IL-4 + IL-13 are in line with the β-3-tubulin^+^ network analysis, which also reported stronger TGF-β effects.

Both TGF-β and IL-4 + IL-13 have previously been implicated in neuroplastic processes; however, our aim was to first demonstrate their effects on peripheral sensory neurons in an *in vitro* type 2 inflammation model. IL-4 and/or IL-13 have been implicated in central nervous system repair and growth (78–80), with IL-4Rα-deficient mice showing impaired synaptic signaling in the central nervous system (81), supporting the idea that these cytokines modulate neuronal connectivity. For the periphery, lung-innervating Dbh^+^ neurons in the nucleus of the solitary tract were activated upon allergen stimulation in an IL-4 dependent manner (82), suggesting that IL-4 is a mediator between allergic inflammation and neuronal activity in airway hyperresponsiveness. Dorsal root ganglia sensory neurons were directly activated by IL-4, IL-13, and IL-31 via IL-4Rα and JAK1 signaling in chronic itch, supporting the sensitization of peripheral sensory neurons by these type 2 cytokines (83). Moreover, JAK1 activation in airway sensory neurons also increases CGRPβ release, resulting in the suppression of ILC2-driven airway inflammation (84). Mouse jugular nodose neurons upregulate NPYR1, which is implicated in neuronal excitability and synaptic communication (85), after IL-1β, Il-13, and BDNF exposure, further supporting the claim that inflammatory cues induce transcriptional changes in sensory neurons (18). TGF-β, known for promoting neuroprotection and synaptic activity in the CNS of mice (86), has limited data on peripheral neurons. Few animal studies have reported enhanced neurite outgrowth and sensitivity, but not in lung-associated neurons (87–89). One study reported enhanced maturation of stem cell-derived neurons in response to TGF-β1, similar to our findings (90). Together, these findings support a model of sensory neuroplasticity through cytokine-driven inflammation, providing a basis for neuroimmune mechanisms in chronic inflammation.

Overall, we found stronger neuroplasticity effects after TGF-β treatment than after IL-4+IL-13 treatment. TGF-β treatment induced neuroplasticity, and transcriptional data showed upregulation of pathways linked to tissue remodeling, including protein, cytoskeleton, and chromatin remodeling, and RNA and DNA metabolism after TGF-β treatment. IL-4 + IL-13 treatment resulted in minimal transcriptional changes related to tissue remodeling and less pronounced neuroplasticity, despite the similar expression of TGF-β and IL-4 + IL-13 receptors. The less dense network and milder hypersensitivity after IL-4 + IL-13 treatment suggest incomplete remodeling after 5-days. Potentially, more complete neuroplasticity could be induced through IL-13 induced TGF-β production through the IL-13α_2_ receptor, as we observed upregulation of IL13RA2 after IL-4 + IL-13 treatment (91). However, the transcriptional downregulation of network formation pathways suggests a complete but weaker neuroplasticity effect of IL-4 + IL-13 than TGF-β. This aligns with the role of TGF-β *in vivo*, where it affects multiple cell types and is strongly linked to airway and ECM remodeling. Although TGF-β alone was sufficient to induce neuroplasticity, IL-4 + IL-13 may require additional interplay with type 2-associated immune cells, their cytokines, or even direct contact (4, 21). Direct contact between sensory neurons and type 2-associated cells, including eosinophils and mast cells, has been previously observed (4, 6). Notably, CADM1, a cell adhesion molecule that mediates direct physical contact between cells, is required to enhance mast cell degranulation (92). This indicates a role for direct neuron-immune contacts in amplifying airway inflammation. Nonetheless, whether IL-4 + IL-13 alone can induce neuroplasticity without direct cell contact remains unclear. Future studies should investigate the inflammation profile of neuroplasticity to identify specific neuro-immune interactions contributing to this pathological feature. Additionally, sensory neuron-immune cell co-cultures should be established to identify mediators of direct cell–cell contact and soluble mediators.

## Conclusion

In conclusion, we developed a robust protocol that enables the differentiation of hPSCs into peripheral sensory neurons. We characterized the sensory neurons using flow cytometry, immunofluorescence, and RNA-sequencing, and established that the generated hPSC-derived neurons exert typical characteristics of sensory neurons, such as the expression of sensory-specific markers TRPV1, Nav1.7, Nav1.8, and CGRP. Furthermore, we found that type 2 cytokines IL-4 + IL-13 and growth factor TGF-β induced neuroplasticity in this human sensory neuron model. The ability to induce neuroplasticity provides an opportunity for future studies to investigate how neuro-immune interactions are involved in the pathological features of neuroplasticity in chronic type 2 inflammation.

## Data Availability

The datasets presented in this study can be found in online repositories. The names of the repository/repositories and accession number(s) can be found below: GSE307973 (GEO).

## Supplementary material

Supplementary Figure 1 Gating strategy for flow cytometry analysis of hPSC-derived sensory neurons. Representative FlowJo gating strategy used to analyze hPSC-derived sensory neurons. Cells were first gated to exclude debris based on FSC/SSC properties, followed by selection of single cells using FSC-A versus FSC-H. Within the singlet population, negative gates were defined for each antibody to establish background fluorescence levels. Final gates show the positive populations for β3-tubulin-PE (and for, ChAT-APC, TRPV1-AF488, Nav1.8-AF488, and Nav1.7-PE – *not shown*). All samples were fixed, permeabilized, stained and measured as described in the Methods, and analysed using FlowJo V10.

Supplementary Figure 2 Multi-electrode measurement show spontaneous firing for mature sensory neurons. Spontaneous firing was detected from around day 30 of the differentiation protocol and continued through day 50. Afterwards, firing was lost in some cases – therefore the optimal timeframe for experiments was determined to be between day 35 and day 50. Statistical significance was tested in comparison to day 22 only using unpaired repeated measures one-way ANOVA with Dunnett’s *post-hoc* correction.

Supplementary Figure 3 Comparing our hPSC-derived sensory neurons to pulmonary sensory neurons. Comparison of our hPSC-derived sensory neurons, to their origin hPSCs (H9WA09), the hPSC-derived cholinergic neurons and two pseudo-bulk RNA-seq datasets of murine dorsal root ganglion nociceptors and murine nodose ganglion nociceptors. Expression was compared for pluripotency markers SOX2, SOX10 and POU5F1, and for a literature-based marker set for pulmonary-specific sensory neurons.

Supplementary Figure 4 Clear expression of TGF-β, IL-4 and IL-13 receptors in hPSC-derived sensory neurons both with and without cytokines treatment. Log2-normalized RNA-seq counts from the DESeq2 analysis plotted for TGF-β (TGFBR1, TGFBR2, TGFBR3, TGFBRAP1, TGFBR3L), IL-4 (IL4R) and IL-13 (IL13RA1, IL13RA2) receptor genes across control (BSA), TGF-β-treated, and IL-4+IL-13-treated conditions. Cytokine exposure did not alter receptor expression levels, except for an increase in IL13RA2 following IL-4+IL-13 treatment.

Supplementary Figure 5 TGF-β treatment induced signaling through SMAD2/3. Geneset Variation Analysis comparing control sensory neurons samples to shows that TGF-β treatment induces signaling through SMAD2/3. With statistical significance indicated if Padj < 0.05 (*) and a trend indicated if 0.1 < Padj < 0.6 (-).

Supplementary Figure 6 hPSC-derived sensory neurons treated with IL-4+IL-13 show transcriptional changes in network-density related pathways. GSEA analysis of IL-4+IL-13 exposure on network density-related biological processes.

Supplementary Figure 7 hPSC-derived sensory neurons treated with IL-4+IL-13 show little transcriptional changes related to sensory neurons hypersensitivity. A] GSEA analysis of IL-4+IL-13 exposure on network sensitivity-related biological processes. B] GSEA analysis of IL-4+IL-13 exposure on ion channels-related biological processes.

Supplementary Video 1 Video of differentiation from vagal neural crest precursor to mature hPSC-derived sensory neurons. Video created with IncuCyte S3 Live-Cell Analysis System, magnification 20x, pictures taken every 2 hrs from day 16 until day 42 of differentiation.

## References

[1] Talbot S Abdulnour REE Burkett PR Lee S Cronin SJF Pascal MA . Silencing nociceptor neurons reduces allergic airway inflammation. Neuron. (2015) 87:341–54. doi: 10.1016/j.neuron.2015.06.007, PMID: 26119026 PMC4506220

[2] Satia I Watson R Scime T Dockry RJ Sen S Ford JW . Allergen challenge increases capsaicin-evoked cough responses in patients with allergic asthma. J Allergy Clin Immunol. (2019) 144:788–95.e1. doi: 10.1016/j.jaci.2018.11.050, PMID: 30660644

[3] Järvikallio A Harvima IT Naukkarinen A . Mast cells, nerves and neuropeptides in atopic dermatitis and nummular eczema. Arch Dermatol Res. (2003) 295:2–7. doi: 10.1007/s00403-002-0378-z, PMID: 12709813

[4] Drake MG Scott GD Blum ED Lebold KM Nie Z Lee JJ . Eosinophils increase airway sensory nerve density in mice and in human asthma. Sci Transl Med. (2018) 10:eaar8477. doi: 10.1126/scitranslmed.aar8477, PMID: 30185653 PMC6592848

[5] Drake MG Cook M Fryer AD Jacoby DB Scott GD . Airway sensory nerve plasticity in asthma and chronic cough. Front Physiol. (2021) 12:720538. doi: 10.3389/fphys.2021.720538, PMID: 34557110 PMC8452850

[6] Dragunas G Koster CS De Souza Xavier Costa N Melgert BN Munhoz CD Gosens R . Neuroplasticity and neuroimmune interactions in fatal asthma. Allergy. (2025) 80:462–73. doi: 10.1111/all.16373, PMID: 39484998

[7] Serhan N Basso L Sibilano R Petitfils C Meixiong J Bonnart C . House dust mites activate nociceptor–mast cell clusters to drive type 2 skin inflammation. Nat Immunol. (2019) 20:1435–43. doi: 10.1038/s41590-019-0493-z, PMID: 31591569 PMC6858877

[8] Tamari M Ver Heul AM . Neuroimmune mechanisms of type 2 inflammation in the skin and lung. Allergol Int. (2025) 74:177–86. doi: 10.1016/j.alit.2025.02.001, PMID: 40064568

[9] Goldsteen PA Sabogal Guaqueta AM Mulder PPMFA Bos IST Eggens M van der Koog L . Differentiation and on axon-guidance chip culture of human pluripotent stem cell-derived peripheral cholinergic neurons for airway neurobiology studies. Front Pharmacol. (2022) 13:991072. doi: 10.3389/fphar.2022.991072, PMID: 36386177 PMC9651921

[10] Kistemaker LEM Prakash YS . Airway innervation and plasticity in asthma. Physiology. (2019) 34:283–98. doi: 10.1152/physiol.00050.2018, PMID: 31165683 PMC6863372

[11] Cramer SC Sur M Dobkin BH O’Brien C Sanger TD Trojanowski JQ . Harnessing neuroplasticity for clinical applications. Brain. (2011) 134:1591–609. doi: 10.1093/brain/awr039, PMID: 21482550 PMC3102236

[12] O’Hanlon S Facer P Simpson KD Sandhu G Saleh HA Anand P . Neuronal markers in allergic rhinitis: expression and correlation with sensory testing. Laryngoscope. (2007) 117:1519–27. doi: 10.1097/MLG.0b013e3180ca7846, PMID: 17667132

[13] Dragunas G Woest ME Nijboer S Bos ST Asselt J Groot AP . Cholinergic neuroplasticity in asthma driven by TrkB signaling. FASEB J. (2020) 34:7703–17. doi: 10.1096/fj.202000170R, PMID: 32277855 PMC7302963

[14] Zaccone EJ Undem BJ . Airway vagal neuroplasticity associated with respiratory viral infections. Lung. (2016) 194:25–9. doi: 10.1007/s00408-015-9832-5, PMID: 26678280 PMC4827158

[15] Pincus AB Huang SJ Lebold KM de la Torre U Proskocil BJ Drake MG . Multicolor labeling of airway neurons and analysis of parasympathetic heterogeneity. Sci Rep. (2022) 12:1–8. doi: 10.1038/s41598-022-08655-6, PMID: 35322058 PMC8943012

[16] Kim SH Patil MJ Hadley SH Bahia PK Butler SG Madaram M . Mapping of the sensory innervation of the mouse lung by specific vagal and dorsal root ganglion neuronal subsets. eneuro. (2022) 9:ENEURO.0026–22.2022. doi: 10.1523/ENEURO.0026-22.2022, PMID: 35365503 PMC9015009

[17] Su Y Barr J Jaquish A Xu J Verheyden JM Sun X . Identification of lung innervating sensory neurons and their target specificity. Am J Physiol-Lung Cell Mol Physiol. (2022) 322:L50–63. doi: 10.1152/ajplung.00376.2021, PMID: 34755535 PMC8721910

[18] Crosson T Bhat S Wang JC Salaun C Fontaine E Roversi K . Cytokines reprogram airway sensory neurons in asthma. Cell Reports. (2024) 43:115045. doi: 10.1016/j.celrep.2024.115045, PMID: 39661516

[19] Chen J Xie S Lin Z Zhao C Tao R Ma Y . Neural circuits between nodose ganglion and pulmonary neuroendocrine cells regulate lung inflammatory responses. Neuroscience. (2025). doi: 10.1101/2025.01.01.630820, PMID: 41610307 PMC12948239

[20] Kulka M Sheen CH Tancowny BP Grammer LC Schleimer RP . Neuropeptides activate human mast cell degranulation and chemokine production. Immunology. (2008) 123:398–410. doi: 10.1111/j.1365-2567.2007.02705.x, PMID: 17922833 PMC2433325

[21] Kornfield JM Bright H Drake MG . Touching a nerve: neuroimmune interactions in asthma. Immunol Rev. (2025) 331:e70025. doi: 10.1111/imr.70025, PMID: 40186378 PMC12121487

[22] Belvisi M . Overview of the innervation of the lung. Curr Opin Pharmacol. (2002) 2:211–5. doi: 10.1016/S1471-4892(02)00145-5, PMID: 12020459

[23] Bevan S Szolcsányi J . Sensory neuron-specific actions of capsaicin_ mechanisms and applications. Trends Pharmacol Sci. (1990) 11:331–3. doi: 10.1016/0165-6147(90)90237-3, PMID: 2203194

[24] Yang F Zheng J . Understand spiciness: mechanism of TRPV1 channel activation by capsaicin. Protein Cell. (2017) 8:169–77. doi: 10.1007/s13238-016-0353-7, PMID: 28044278 PMC5326624

[25] León B Ballesteros-Tato A . Modulating Th2 cell immunity for the treatment of asthma. Front Immunol. (2021) 12:637948. doi: 10.3389/fimmu.2021.637948, PMID: 33643321 PMC7902894

[26] Ricciardolo FLM Sprio AE Baroso A Gallo F Riccardi E Bertolini F . Characterization of T2-low and T2-high asthma phenotypes in real-life. Biomedicines. (2021) 9:1684. doi: 10.3390/biomedicines9111684, PMID: 34829913 PMC8615363

[27] Ottosson A Edvinsson L . Release of histamine from dural mast cells by substance P and calcitonin gene-related peptide. Cephalalgia. (1997) 17:166–74. doi: 10.1046/j.1468-2982.1997.1703166.x, PMID: 9170339

[28] Weidner C Klede M Rukwied R Lischetzki G Neisius U Schmelz M . Acute effects of substance P and calcitonin gene-related peptide in human skin – A microdialysis study. J Invest Dermatol. (2000) 115:1015–20. doi: 10.1046/j.1523-1747.2000.00142.x, PMID: 11121135

[29] Ollerenshaw S Jarvis D Sullivan C Woolcock A . Substance P immunoreactive nerves in airways from asthmatics and nonasthmatics. Eur Respir J. (1991) 4:673–82. doi: 10.1183/09031936.93.04060673, PMID: 1716217

[30] Klein Wolterink RGJ Wu GS Chiu IM Veiga-Fernandes H . Neuroimmune interactions in peripheral organs. Annu Rev Neurosci. (2022) 45:339–60. doi: 10.1146/annurev-neuro-111020-105359, PMID: 35363534 PMC9436268

[31] Basso L Serhan N Tauber M Gaudenzio N . Peripheral neurons: Master regulators of skin and mucosal immune response. Eur J Immunol. (2019) 49:1984–97. doi: 10.1002/eji.201848027, PMID: 31327163

[32] Carpentier G. Berndt S. Ferratge S. Rasband W. Cuendet M. Uzan G. . Angiogenesis Analyzer for ImageJ — A comparative morphometric analysis of “Endothelial Tube Formation Assay” and “Fibrin Bead Assay”. Sci Rep. (2020) 10:11568. doi: 10.1038/s41598-020-67289-8, PMID: 32665552 PMC7360583

[33] Monaco G Chen H Poidinger M Chen J De Magalhães JP Larbi A . flowAI: automatic and interactive anomaly discerning tools for flow cytometry data. Bioinformatics. (2016) 32:2473–80. doi: 10.1093/bioinformatics/btw191, PMID: 27153628

[34] Love MI Huber W Anders S . Moderated estimation of fold change and dispersion for RNA-seq data with DESeq2. Genome Biol. (2014) 15:550. doi: 10.1186/s13059-014-0550-8, PMID: 25516281 PMC4302049

[35] Zhu A Ibrahim JG Love MI . Heavy-tailed prior distributions for sequence count data: removing the noise and preserving large differences. Bioinformatics. (2019) 35:2084–92. doi: 10.1093/bioinformatics/bty895, PMID: 30395178 PMC6581436

[36] Korotkevich G Sukhov V Budin N Shpak B Artyomov MN Sergushichev A . Fast gene set enrichment analysis. bioRxiv. (2016) 060012. doi: 10.1101/060012

[37] De Virgiliis F Di Giovanni S . Lung innervation in the eye of a cytokine storm: neuroimmune interactions and COVID-19. Nat Rev Neurol. (2020) 16:645–52. doi: 10.1038/s41582-020-0402-y, PMID: 32843733 PMC7446605

[38] Chang RB Strochlic DE Williams EK Umans BD Liberles SD . Vagal sensory neuron subtypes that differentially control breathing. Cell. (2015) 161:622–33. doi: 10.1016/j.cell.2015.03.022, PMID: 25892222 PMC4842319

[39] Nickolls AR Lee MM Espinoza DF Szczot M Lam RM Wang Q . Transcriptional programming of human mechanosensory neuron subtypes from pluripotent stem cells. Cell Rep. (2020) 30:932–46.e7. doi: 10.1016/j.celrep.2019.12.062, PMID: 31968264 PMC7059559

[40] Lyoo KS Kim HM Lee B Che YH Kim SJ Song D . Direct neuronal infection of SARS-CoV-2 reveals cellular and molecular pathology of chemosensory impairment of COVID-19 patients. Emerg Microbes Infect. (2022) 11:407–12. doi: 10.1080/22221751.2021.2024095, PMID: 34962444 PMC8803065

[41] May-Zhang AA Tycksen E Southard-Smith AN Deal KK Benthal JT Buehler DP . Combinatorial transcriptional profiling of mouse and human enteric neurons identifies shared and disparate subtypes *in situ*. Gastroenterology. (2021) 160:755–70.e26. doi: 10.1053/j.gastro.2020.09.032, PMID: 33010250 PMC7878294

[42] Hoelting L Klima S Karreman C Grinberg M Meisig J Henry M . Stem cell-derived immature human dorsal root ganglia neurons to identify peripheral neurotoxicants. Stem Cells Transl Med. (2016) 5:476–87. doi: 10.5966/sctm.2015-0108, PMID: 26933043 PMC4798731

[43] Raphael I Nalawade S Eagar TN Forsthuber TG . T cell subsets and their signature cytokines in autoimmune and inflammatory diseases. Cytokine. (2015) 74:5–17. doi: 10.1016/j.cyto.2014.09.011, PMID: 25458968 PMC4416069

[44] Frøssing L Silberbrandt A Von Bülow A Backer V Porsbjerg C . The prevalence of subtypes of type 2 inflammation in an unselected population of patients with severe asthma. J Allergy Clin Immunol Pract. (2021) 9:1267–75. doi: 10.1016/j.jaip.2020.09.051, PMID: 33039645

[45] Choy DF Modrek B Abbas AR Kummerfeld S Clark HF Wu LC . Gene expression patterns of Th2 inflammation and intercellular communication in asthmatic airways. J Immunol Baltim Md 1950. (2011) 186:1861–9. doi: 10.4049/jimmunol.1002568, PMID: 21187436 PMC3981556

[46] Hammad H Lambrecht BN . The basic immunology of asthma. Cell. (2021) 184:1469–85. doi: 10.1016/j.cell.2021.02.016, PMID: 33711259

[47] Al-Alawi M Hassan T Chotirmall SH . Transforming growth factor β and severe asthma: A perfect storm. Respir Med. (2014) 108:1409–23. doi: 10.1016/j.rmed.2014.08.008, PMID: 25240764

[48] Chomarat P Banchereau J . Interleukin-4 and lnterleukin-13: their similarities and discrepancies. Int Rev Immunol. (1998) 17:1–52. doi: 10.3109/08830189809084486, PMID: 9914942

[49] Tsantoulas C McMahon SB . Opening paths to novel analgesics: the role of potassium channels in chronic pain. Trends Neurosci. (2014) 37:146–58. doi: 10.1016/j.tins.2013.12.002, PMID: 24461875 PMC3945816

[50] Rienecker KDA Poston RG Saha RN . Merits and limitations of studying neuronal depolarization-dependent processes using elevated external potassium. ASN Neuro. (2020) 12:1759091420974807. doi: 10.1177/1759091420974807, PMID: 33256465 PMC7711227

[51] Lutz W Sułkowski WJ . Vagus nerve participates in the regulation of the airways: inflammatory response and hyperreactivity induced by occupational asthmogens. Int J Occup Med Environ Health. (2004) 17:417–31. 15852756

[52] Kornfield J de la Torre U Mize E Drake MG . Illuminating airway nerve structure and function in chronic cough. Lung. (2023) 201:499–509. doi: 10.1007/s00408-023-00659-x, PMID: 37985513 PMC10673771

[53] Yasuhara O Aimi Y Shibano A Kimura H . Primary sensory neurons containing choline acetyltransferase of the peripheral type in the rat trigeminal ganglion and their relation to neuropeptides-, calbindin- and nitric oxide synthase-containing cells. Brain Res. (2007) 1141:92–8. doi: 10.1016/j.brainres.2007.01.025, PMID: 17291466

[54] Ehrhardt E Boyan G . Evidence for the cholinergic markers ChAT and vAChT in sensory cells of the developing antennal nervous system of the desert locust Schistocerca gregaria. Invert Neurosci. (2020) 20:19. doi: 10.1007/s10158-020-00252-4, PMID: 33090291 PMC7581592

[55] Yasuyama K Salvaterra PM . Localization of choline acetyltransferase-expressing neurons inDrosophila nervous system. Microsc Res Tech. (1999) 45:65–79. doi: 10.1002/(SICI)1097-0029(19990415)45:2<65::AID-JEMT2>3.0.CO;2-0 10332725

[56] Corsetti V Perrone-Capano C Salazar Intriago MS Botticelli E Poiana G Augusti-Tocco G . Expression of cholinergic markers and characterization of splice variants during ontogenesis of rat dorsal root ganglia neurons. Int J Mol Sci. (2021) 22:5499. doi: 10.3390/ijms22115499, PMID: 34071104 PMC8197147

[57] Plumbly W Patikas N Field SF Foskolou S Metzakopian E . Derivation of nociceptive sensory neurons from hiPSCs with early patterning and temporally controlled NEUROG2 overexpression. Cell Rep Methods. (2022) 2:100341. doi: 10.1016/j.crmeth.2022.100341, PMID: 36452863 PMC9701618

[58] Deng T Jovanovic VM Tristan CA Weber C Chu PH Inman J . Scalable generation of sensory neurons from human pluripotent stem cells. Stem Cell Rep. (2023) 18:1030–47. doi: 10.1016/j.stemcr.2023.03.006, PMID: 37044067 PMC10147831

[59] Guimarães MZP De Vecchi R Vitória G Sochacki JK Paulsen BS Lima I . Generation of iPSC-derived human peripheral sensory neurons releasing substance P elicited by TRPV1 agonists. Front Mol Neurosci. (2018) 11:277. doi: 10.3389/fnmol.2018.00277, PMID: 30186108 PMC6113370

[60] Ogawa T Yamada S Fukushi S Imai Y Kawada J Ikeda K . Formation and long-term culture of hiPSC-derived sensory nerve organoids using microfluidic devices. Bioengineering. (2024) 11:794. doi: 10.3390/bioengineering11080794, PMID: 39199753 PMC11352057

[61] Moosa A LeBlanc N Louis SA Eaves AC Knock E . Efﬁcient Differentiation of Human Pluripotent Stem Cells to Sensory Neurons. Available online at: https://cdn.stemcell.com/media/files/poster/SP00215-Efficient_Generation_of_Human_Pluripotent_Stem_Cells_to_Neural_Crest_Cells.pdf.

[62] Fischer MJM Ciotu CI Szallasi A . The mysteries of capsaicin-sensitive afferents. Front Physiol. (2020) 11:554195/full. doi: 10.3389/fphys.2020.554195/full 33391007 PMC7772409

[63] Odawara A Shibata M Ishibashi Y Nagafuku N Matsuda N Suzuki I . *In vitro* pain assay using human iPSC-derived sensory neurons and microelectrode array. Toxicol Sci. (2022) 188:131–41. doi: 10.1093/toxsci/kfac045, PMID: 35478041

[64] Hiranuma M Okuda Y Fujii Y Richard JP Watanabe T . Characterization of human iPSC-derived sensory neurons and their functional assessment using multi electrode array. Sci Rep. (2024) 14:6011. doi: 10.1038/s41598-024-55602-8, PMID: 38472288 PMC10933446

[65] Kraik K Tota M Laska J Łacwik J Paździerz Ł Sędek Ł . The role of transforming growth factor-β (TGF-β) in asthma and chronic obstructive pulmonary disease (COPD). Cells. (2024) 13:1271. doi: 10.3390/cells13151271, PMID: 39120302 PMC11311642

[66] Bagnasco D Ferrando M Varricchi G Passalacqua G Canonica GW . A critical evaluation of anti-IL-13 and anti-IL-4 strategies in severe asthma. Int Arch Allergy Immunol. (2016) 170:122–31. doi: 10.1159/000447692, PMID: 27637004

[67] Lindhout FW Portegies S Kooistra R Herstel LJ Stucchi R Hummel JJA . Centrosome-mediated microtubule remodeling during axon formation in human iPSC-derived neurons. EMBO J. (2021) 40:e106798. doi: 10.15252/embj.2020106798, PMID: 33835529 PMC8126955

[68] Gradari S Herrera A Tezanos P Fontán-Lozano Á Pons S Trejo JL . The role of Smad2 in adult neuroplasticity as seen through hippocampal-dependent spatial learning/memory and neurogenesis. J Neurosci. (2021) 41:6836–49. doi: 10.1523/JNEUROSCI.2619-20.2021, PMID: 34210778 PMC8360684

[69] Hiew LF Poon CH You HZ Lim LW . TGF-β/Smad signalling in neurogenesis: implications for neuropsychiatric diseases. Cells. (2021) 10:1382. doi: 10.3390/cells10061382, PMID: 34205102 PMC8226492

[70] Melchionna R Trono P Tocci A Nisticò P . Actin cytoskeleton and regulation of TGFβ Signaling: exploring their links. Biomolecules. (2021) 11:336. doi: 10.3390/biom11020336, PMID: 33672325 PMC7926735

[71] Verrecchia F Mauviel A . Transforming growth factor-β Signaling through the Smad pathway: role in extracellular matrix gene expression and regulation. J Invest Dermatol. (2002) 118:211–5. doi: 10.1046/j.1523-1747.2002.01641.x, PMID: 11841535

[72] Deng Z Fan T Xiao C Tian H Zheng Y Li C . TGF-β signaling in health, disease and therapeutics. Signal Transduct Target Ther. (2024) 9:61. doi: 10.1038/s41392-024-01764-w, PMID: 38514615 PMC10958066

[73] Zhang YE . Non-Smad signaling pathways of the TGF-β Family. Cold Spring Harb Perspect Biol. (2017) 9:a022129. doi: 10.1101/cshperspect.a022129, PMID: 27864313 PMC5287080

[74] Guan G Cannon RD Coates DE Mei L . Effect of the Rho-kinase/ROCK signaling pathway on cytoskeleton components. Genes. (2023) 14:272. doi: 10.3390/genes14020272, PMID: 36833199 PMC9957420

[75] Walters GC Usachev YM . Mitochondrial calcium cycling in neuronal function and neurodegeneration. Front Cell Dev Biol. (2023) 11:1094356. doi: 10.3389/fcell.2023.1094356, PMID: 36760367 PMC9902777

[76] Chlebanowska P Szlaga A Tejchman-Skrzyszewska A Kot M Konieczny P Skrzypek K . Mitochondrial fitness influences neuronal excitability of dopaminergic neurons from patients with idiopathic form of Parkinson’s disease. bioRxiv. (2023) 2023.04.28.538698. doi: 10.1101/2023.04.28.538698, PMID: 41723302

[77] Kim DS Choi JO Rim HD Cho HJ . Downregulation of voltage-gated potassium channel α gene expression in dorsal root ganglia following chronic constriction injury of the rat sciatic nerve. Mol Brain Res. (2002) 105:146–52. doi: 10.1016/S0169-328X(02)00388-1, PMID: 12399117

[78] Jeong JY Chung YC Jin BK . Interleukin-4 and interleukin-13 exacerbate neurotoxicity of prothrombin kringle-2 in cortex *in vivo* via oxidative stress. Int J Mol Sci. (2019) 20:1927. doi: 10.3390/ijms20081927, PMID: 31010119 PMC6515094

[79] Mashkaryan V Siddiqui T Popova S Cosacak MI Bhattarai P Brandt K . Type 1 Interleukin-4 Signaling Obliterates Mouse Astroglia *in vivo* but Not *in vitro*. Front Cell Dev Biol. (2020) 8:114. doi: 10.3389/fcell.2020.00114, PMID: 32181251 PMC7057913

[80] Li S Olde Heuvel F Rehman R Aousji O Froehlich A Li Z . Interleukin-13 and its receptor are synaptic proteins involved in plasticity and neuroprotection. Nat Commun. (2023) 14:200. doi: 10.1038/s41467-023-35806-8, PMID: 36639371 PMC9839781

[81] Hanuscheck N Thalman C Domingues M Schmaul S Muthuraman M Hetsch F . Interleukin-4 receptor signaling modulates neuronal network activity. J Exp Med. (2022) 219:e20211887. doi: 10.1084/jem.20211887, PMID: 35587822 PMC9123307

[82] Su Y Xu J Zhu Z Chin J Xu L Yu H . Brainstem Dbh+ neurons control allergen-induced airway hyperreactivity. Nature. (2024) 631:601–9. doi: 10.1038/s41586-024-07608-5, PMID: 38987587 PMC11254774

[83] Oetjen LK Mack MR Feng J Whelan TM Niu H Guo CJ . Sensory neurons co-opt classical immune signaling pathways to mediate chronic itch. Cell. (2017) 171:217–28.e13. doi: 10.1016/j.cell.2017.08.006, PMID: 28890086 PMC5658016

[84] Tamari M Del Bel KL Ver Heul AM Zamidar L Orimo K Hoshi M . Sensory neurons promote immune homeostasis in the lung. Cell. (2024) 187:44–61.e17. doi: 10.1016/j.cell.2023.11.027, PMID: 38134932 PMC10811756

[85] Nelson TS Fu W Donahue RR Corder GF Hökfelt T Wiley RG . Facilitation of neuropathic pain by the NPY Y1 receptor-expressing subpopulation of excitatory interneurons in the dorsal horn. Sci Rep. (2019) 9. doi: 10.1038/s41598-019-43493-z, PMID: 31076578 PMC6510760

[86] Caraci F Gulisano W Guida CA Impellizzeri AAR Drago F Puzzo D . A key role for TGF-β1 in hippocampal synaptic plasticity and memory. Sci Rep. (2015) 5:11252. doi: 10.1038/srep11252, PMID: 26059637 PMC4462026

[87] Zhu Y Colak T Shenoy M Liu L Mehta K Pai R . Transforming growth factor beta induces sensory neuronal hyperexcitability, and contributes to pancreatic pain and hyperalgesia in rats with chronic pancreatitis. Mol Pain. (2012) 8:1744-8069-8-65. doi: 10.1186/1744-8069-8-65, PMID: 22963239 PMC3515355

[88] Wang Y Zhao X Huojia M Xu H Zhuang Y . Transforming growth factor-β3 promotes facial nerve injury repair in rabbits. Exp Ther Med. (2016) 11:703–8. doi: 10.3892/etm.2016.2972, PMID: 26997982 PMC4774367

[89] Jiang M Ding Z Huang Y Jiang T Xia Y Gu D . TGF-β1 improves nerve regeneration and functional recovery after sciatic nerve injury by alleviating inflammation. Biomedicines. (2025) 13:872. doi: 10.3390/biomedicines13040872, PMID: 40299436 PMC12024759

[90] Izsak J Vizlin-Hodzic D Iljin M Strandberg J Jadasz J Olsson Bontell T . TGF-β1 Suppresses Proliferation and Induces Differentiation in Human iPSC Neural *in vitro* Models. Front Cell Dev Biol. (2020) 8:571332. doi: 10.3389/fcell.2020.571332, PMID: 33195202 PMC7655796

[91] Fichtner-Feigl S Strober W Kawakami K Puri RK Kitani A . IL-13 signaling through the IL-13α2 receptor is involved in induction of TGF-β1 production and fibrosis. Nat Med. (2006) 12:99–106. doi: 10.1038/nm1332, PMID: 16327802

[92] Magadmi R Meszaros J Damanhouri ZA Seward EP . Secretion of mast cell inflammatory mediators is enhanced by CADM1-dependent adhesion to sensory neurons. Front Cell Neurosci. (2019) 13:262. doi: 10.3389/fncel.2019.00262, PMID: 31275114 PMC6591473

