# Supplementary figures and images for: TGF-β and IL-4 + IL-13 induce neuroplasticity in an in vitro model of hPSC-derived sensory neurons
Source: Front Immunol. 2026 Mar 3;17:1705880. doi: 10.3389/fimmu.2026.1705880 (PMC12992014; doi:10.3389/fimmu.2026.1705880)

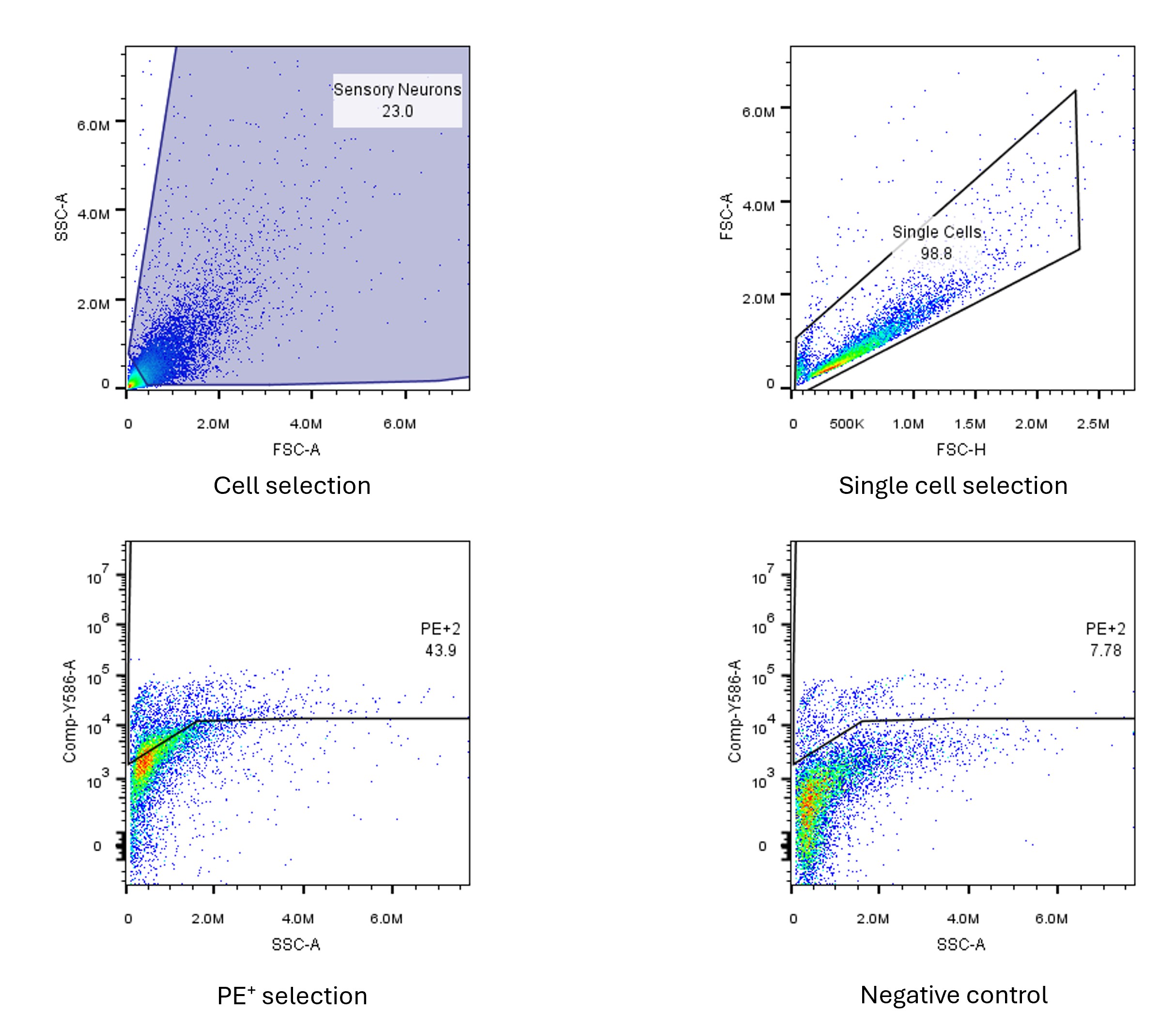

Supplement: Supplementary Figure 1 — Gating strategy for flow cytometry analysis of hPSC-derived sensory neurons. Representative FlowJo gating strategy used to analyze hPSC-derived sensory neurons. Cells were first gated to exclude debris based on FSC/SSC properties, followed by selection of single cells using FSC-A versus FSC-H. Within the singlet population, negative gates were defined for each antibody to establish background fluorescence levels. Final gates show the positive populations for β3-tubulin-PE (and for, ChAT-APC, TRPV1-AF488, Nav1.8-AF488, and Nav1.7-PE – not shown). All samples were fixed, permeabilized, stained and measured as described in the Methods, and analysed using FlowJo V10. [file Image1.jpeg]

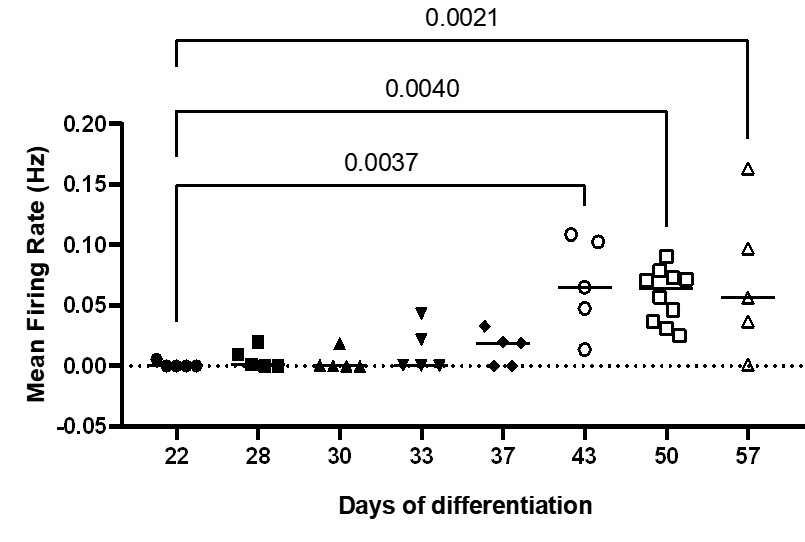

Supplement: Supplementary Figure 2 — Multi-electrode measurement show spontaneous firing for mature sensory neurons. Spontaneous firing was detected from around day 30 of the differentiation protocol and continued through day 50. Afterwards, firing was lost in some cases – therefore the optimal timeframe for experiments was determined to be between day 35 and day 50. Statistical significance was tested in comparison to day 22 only using unpaired repeated measures one-way ANOVA with Dunnett’s post-hoc correction. [file Image2.jpeg]

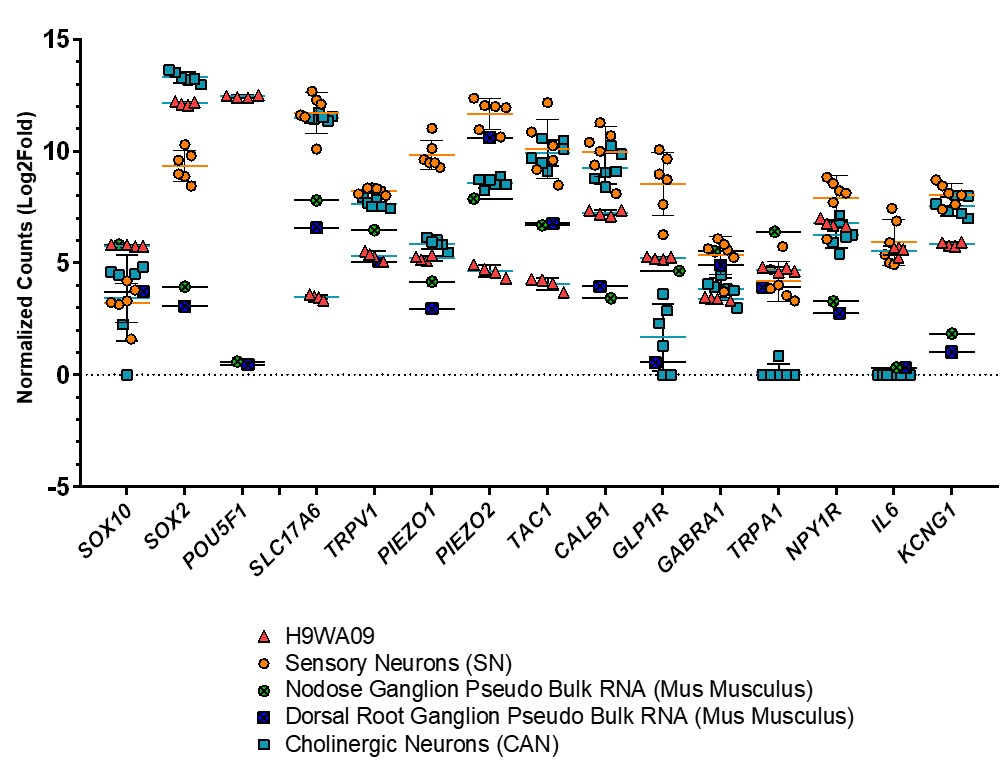

Supplement: Supplementary Figure 3 — Comparing our hPSC-derived sensory neurons to pulmonary sensory neurons. Comparison of our hPSC-derived sensory neurons, to their origin hPSCs (H9WA09), the hPSC-derived cholinergic neurons and two pseudo-bulk RNA-seq datasets of murine dorsal root ganglion nociceptors and murine nodose ganglion nociceptors. Expression was compared for pluripotency markers SOX2, SOX10 and POU5F1, and for a literature-based marker set for pulmonary-specific sensory neurons. [file Image3.jpeg]

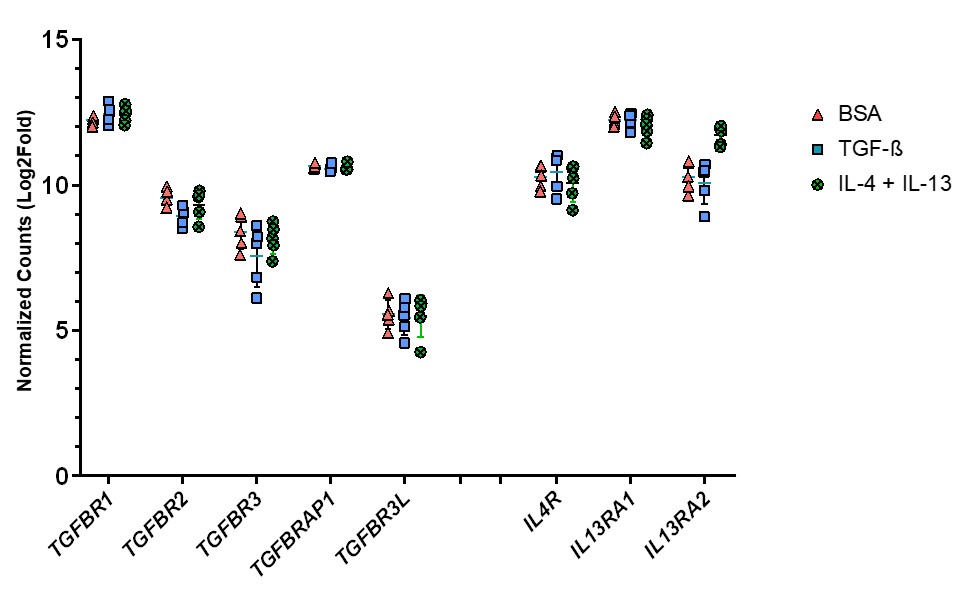

Supplement: Supplementary Figure 4 — Clear expression of TGF-β, IL-4 and IL-13 receptors in hPSC-derived sensory neurons both with and without cytokines treatment. Log2-normalized RNA-seq counts from the DESeq2 analysis plotted for TGF-β (TGFBR1, TGFBR2, TGFBR3, TGFBRAP1, TGFBR3L), IL-4 (IL4R) and IL-13 (IL13RA1, IL13RA2) receptor genes across control (BSA), TGF-β-treated, and IL-4+IL-13-treated conditions. Cytokine exposure did not alter receptor expression levels, except for an increase in IL13RA2 following IL-4+IL-13 treatment. [file Image4.jpeg]

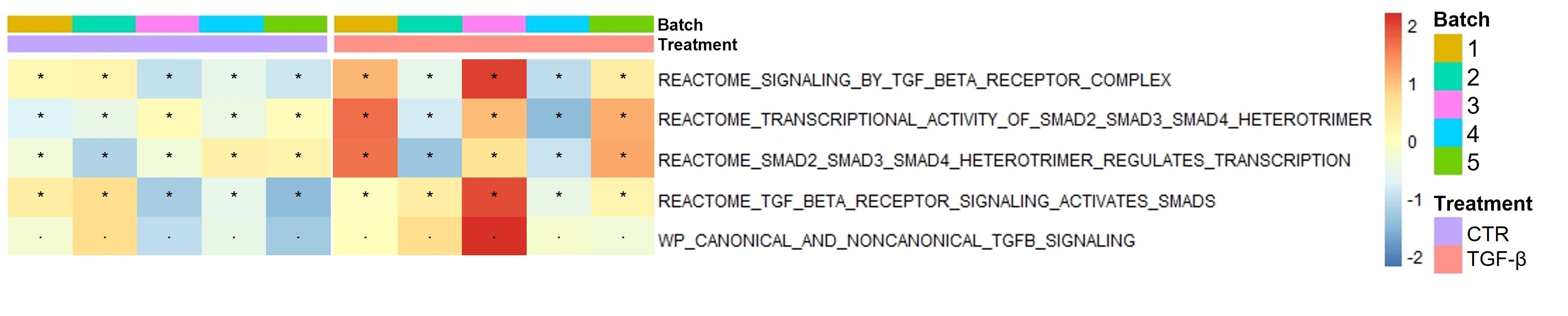

Supplement: Supplementary Figure 5 — TGF-β treatment induced signaling through SMAD2/3. Geneset Variation Analysis comparing control sensory neurons samples to shows that TGF-β treatment induces signaling through SMAD2/3. With statistical significance indicated if Padj < 0.05 (*) and a trend indicated if 0.1 < Padj < 0.6 (-). [file Image5.jpg]

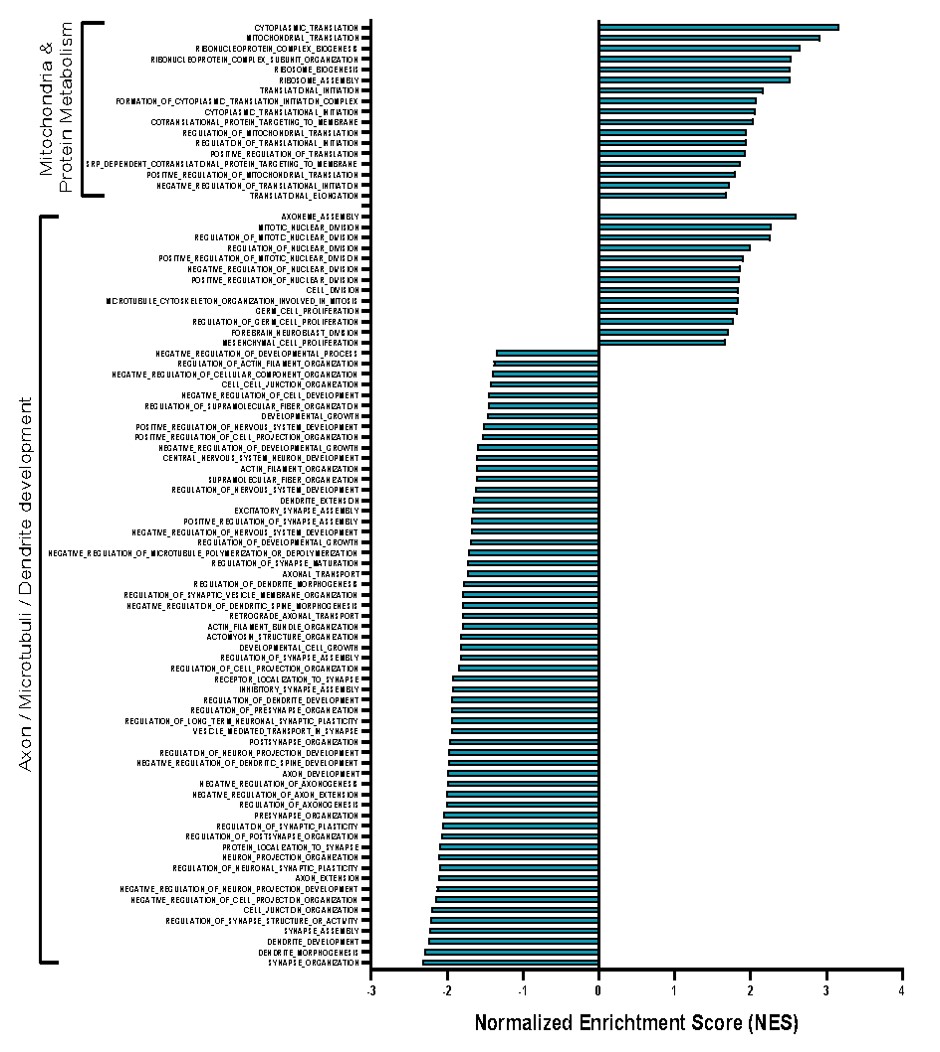

Supplement: Supplementary Figure 6 — hPSC-derived sensory neurons treated with IL-4+IL-13 show transcriptional changes in network-density related pathways. GSEA analysis of IL-4+IL-13 exposure on network density-related biological processes. [file Image6.jpeg]

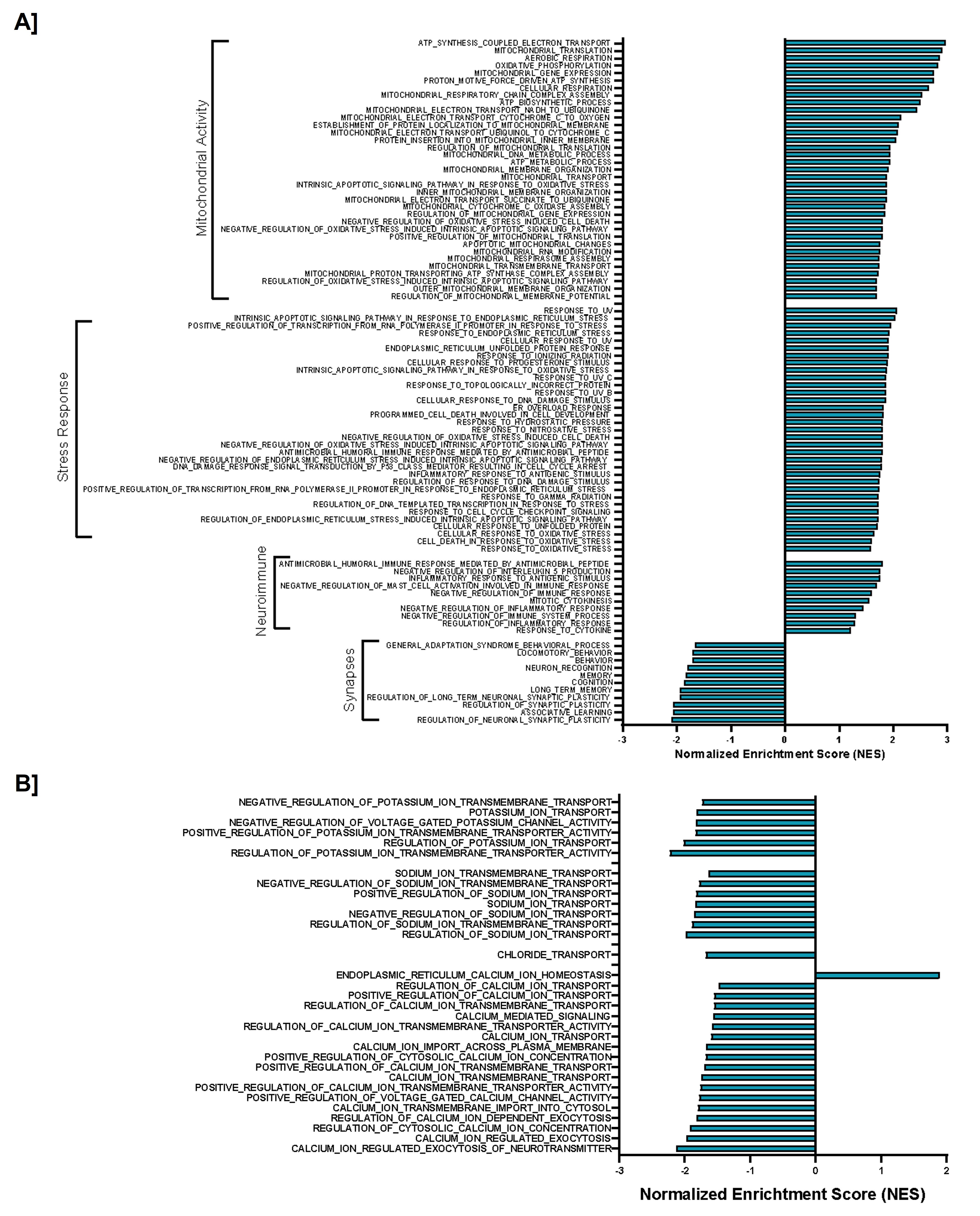

Supplement: Supplementary Figure 7 — hPSC-derived sensory neurons treated with IL-4+IL-13 show little transcriptional changes related to sensory neurons hypersensitivity. A] GSEA analysis of IL-4+IL-13 exposure on network sensitivity-related biological processes. B] GSEA analysis of IL-4+IL-13 exposure on ion channels-related biological processes. [file Image7.jpeg]
