# Supplementary material for: TGF-β and IL-4 + IL-13 induce neuroplasticity in an in vitro model of hPSC-derived sensory neurons
Source: Front Immunol. 2026 Mar 3;17:1705880. doi: 10.3389/fimmu.2026.1705880 (PMC12992014; doi:10.3389/fimmu.2026.1705880)
Supplement: Supplementary file 9 [file Table1.pdf]

Table S1. Antibodies and their respective dilutions and suppliers as used for immunofluorescence.

Antibody	Dilution	Host	Supplier (cat.)
**Anti-β3-Tubulin**	1:200	Mouse monoclonal	Santa Cruz Biotechnology, Inc. (sc-8005)
**Anti-PGP9.5**	1:500	Rabbit	GeneTEx (GTX109637)
**Anti-TRPV1**	1:100 – 1:500	Rabbit	ThermoFisher (PA1-748)
**Anti-Nav1.8 (SCN10A)**	1:300	Rabbit polyclonal	LSBio (LS-C803551-100)
**Anti-VAChT**	1:200	Guinea pig	Synaptic Systems (139105)
**Alexa Fluor 488**	1:1000	Donkey-anti-Mouse	ThermoFisher (A-21202)
**Alexa Fluor 488**	1:1000	Donkey-anti-Rabbit	ThermoFisher (A-21206)
**Alexa Fluor 568**	1:1000	Donkey-anti-Rabbit	ThermoFisher (A-10042)
**Alexa Fluor 568**	1:1000	Donkey-anti-Mouse	ThermoFisher (A-10037)
**Cy™3 AffiniPure**	1:200	Donkey-anti-Guinea Pig	Jackson ImmunoResearch (706-165-148)
