# Supplementary material for: TGF-β and IL-4 + IL-13 induce neuroplasticity in an in vitro model of hPSC-derived sensory neurons
Source: Front Immunol. 2026 Mar 3;17:1705880. doi: 10.3389/fimmu.2026.1705880 (PMC12992014; doi:10.3389/fimmu.2026.1705880)
Supplement: Supplementary file 10 [file Table2.pdf]

Table S2. Antibodies and their respective dilutions and suppliers as used for flow cytometry.

Antibody	Dilution	Host	Supplier (cat.)
**PE Anti-β3-Tubulin**	1:1000	Mouse monoclonal	Biotechne (NB600-1018PE)
**APC Anti-Choline Acetyltransferase**	1:3000	Anti-Human	Abcam (ab224001)
**Anti-TRPV1**	1:500	Rabbit	ThermoFisher (PA1-748)
**Anti-Nav1.8 (SCN10A)**	1:300	Rabbit polyclonal	LSBio (LS-C803551-100)
**Biotin Anti-Nav1.7**	1:50	Guinea pig	Synaptic Systems (139105)
**Alexa Fluor 488**	1:1000	Donkey-anti-Rabbit	ThermoFisher (A-21206)
**PE Streptavidin**	1:100	-	BioLegend (#405203)
