# Supplementary material for: TGF-β and IL-4 + IL-13 induce neuroplasticity in an in vitro model of hPSC-derived sensory neurons
Source: Front Immunol. 2026 Mar 3;17:1705880. doi: 10.3389/fimmu.2026.1705880 (PMC12992014; doi:10.3389/fimmu.2026.1705880)
Supplement: Supplementary file 11 [file Table3.pdf]

Table S3. Overview of primers used for qPCR of human genes.

Gene	Forward primer	Reverse primer
GAPDH	CCA GCA AGA GCA CAA GAG GA	GAG ATT CAG TGT GGT GGG GG
RPL13A	TCG TAC GCT GTG AAG GCA TC	GCT TTT TCT TGT CGT AGG GGG
SDHA	TGC AGA AGG TGC GGA TTG AT	TCC AGA GTG ACC TTC CCA GT
OCT4	GGC CAC ACG TAG GTT CTT GA	GCT GAA TAC CTT CCC AAA TAG AAC C
SOX10	GAC CAG TAC CCG CAC CTG	CGC TTG TCA CTT TCG TTC AG
B3TUB	GGA GAT CGT GCA CAT CCA GG	GCC CCA CTC TGA CCA AAG AT
UCHL1 (PGP9.5)	TTC CTG TGG CAC AAT CGG AC	CAT CTA CCC GAC ATT GGC CT
TAC1	TTA CTG GTC CGA CTG GTA CGA C	TTA CTG GTC CGA CTG GTA CGA C
SCN9A (NAV1.7)	GGT TTC AGC ACA GAT TCA GGT C	CCA GCT GAA AGT GTC AAA GCT C
TRPV1	GGC TGT CTT CAT CAT CCT GCT GCT	GTT CTT GCT CTC CTG TGC GAT CTT GT
VAChT	GTG CGC CAT GTC TCA GTC TA	CTC ATC AAG CAG CAC ATC GC
