# Supplementary material for: TGF-β and IL-4 + IL-13 induce neuroplasticity in an in vitro model of hPSC-derived sensory neurons
Source: Front Immunol. 2026 Mar 3;17:1705880. doi: 10.3389/fimmu.2026.1705880 (PMC12992014; doi:10.3389/fimmu.2026.1705880)
Supplement: Supplementary file 12 [file Table4.pdf]

Table S4. Extraction of the top 50 genes influencing the separation of TGF-β or IL-4+IL-13 treated SN compared to control in principal component 4 and 5.

	PC4	PC5
1	ALOX15	RPL36A-HNRNPH2
2	MME	RNF139
3	CCL26	ALOX15
4	CSN1S1	NewGene_4696
5	CISH	ID3
6	SERPINB11	NewGene_18650
7	UBE2A	ISY1-RAB43
8	PLA2G4B	NewGene_109
9	JMJD7-PLA2G4B	NewGene_9522
10	TMEM71	PCDHAC1
11	COL14A1	MED10
12	ZNF660-ZNF197	ZDBF2
13	APOLD1	PCDHAC2
14	SMR3B	AK6
15	MMP28	ARHGAP36
16	NewGene_6397	GNG4
17	NKRF	GPR50
18	CD44	CARTPT
19	HHIP	ITIH2
20	FGL2	NewGene_18033
21	SLC26A4	NDUFC2-KCTD14
22	OLFML2A	PFDN5
23	FGG	TNFAIP6
24	MSANTD3-TMEFF1	PEDS1-UBE2V1
25	CYSLTR2	NewGene_11866
26	GNG4	CCL26
27	VCAM1	REN
28	COL6A5	DUSP11
29	CAVIN2	NewGene_18879
30	TNFSF10	NewGene_344
31	NewGene_18879	SAA1
32	SERPINB4	POSTN
33	TNFAIP6	NewGene_2577
34	COL6A6	NewGene_15358
35	RHOU	COL10A1
36	POSTN	DBH
37	ITGA11	C11orf91

38	NABP1	GNRH1
39	CLDN5	NewGene_19674
40	CTSV	ENPP1
41	MS4A6A	NewGene_10919
42	HMGA2	ASPN
43	CXCL6	NewGene_16285
44	NewGene_14322	TSC22D4-C7ORF61
45	R3HDML	NewGene_3309
46	NewGene_18033	CTDNEP1
47	IL6	MOSMO
48	TPH1	PRR5-ARHGAP8
49	KCNJ2	NewGene_16275
50	IL13RA2	NewGene_8318
